# Chiral molecular nanosilicas†

**DOI:** 10.1039/d2sc00793b

**Published:** 2022-03-14

**Authors:** Zhaohui Zong, Aiyou Hao, Pengyao Xing, Yanli Zhao

**Affiliations:** Key Laboratory of Colloid and Interface Chemistry of Ministry of Education, School of Chemistry and Chemical Engineering, Shandong University Jinan 250100 People's Republic of China xingpengyao@sdu.edu.cn; Division of Chemistry and Biological Chemistry, School of Physical and Mathematical Sciences, Nanyang Technological University 21 Nanyang Link Singapore 637371 Singapore zhaoyanli@ntu.edu.sg

## Abstract

Molecular nanoparticles including polyoxometalates, proteins, fullerenes and polyhedral oligosiloxane (POSS) are nanosized objects with atomic precision, among which POSS derivatives are the smallest nanosilicas. Incorporation of molecular nanoparticles into chiral aggregates either by chiral matrices or self-assembly allows for the transfer of supramolecular chirality, yet the construction of intrinsic chirality with atomic precision in discrete molecules remains a great challenge. In this work, we present a molecular folding strategy to construct giant POSS molecules with inherent chirality. Ferrocenyl diamino acids are conjugated by two or four POSS segments. Hydrogen bonding-driven folding of diamino acid arms into parallel β-sheets facilitates the chirality transfer from amino acids to ferrocene and POSS respectively, disregarding the flexible alkyl spacers. Single crystal X-ray structures, density functional theory (DFT) calculations, circular dichroism and vibrational circular dichroism spectroscopy clearly verify the preferential formation of one enantiomer containing chiral molecular nanosilicas. The chiral orientation and chiroptical properties of POSS show pronounced dependence on the substituents of α-amino acids, affording an alternative way to control the folding behavior and POSS chirality in addition to the absolute configuration of amino acids. Through the kinetic nanoprecipitation protocol, one-dimensional aggregation enables chirality transfer from the molecular scale to the micrometer scale, self-assembling into helices in accordance with the packing propensity of POSS in a crystal phase. This work, by illustrating the construction of chiral molecular nanosilicas, paves a new way to obtain discrete chiral molecular nanoparticles for potential chiroptical applications.

## Abstract 2

A molecular folding strategy is developed to construct ferrocenyl diamino acid conjugated polyhedral oligosiloxane molecules. Hydrogen bonding-driven folding facilitates the chirality transfer from the molecular scale to the micrometer scale.

## Graphical abstract

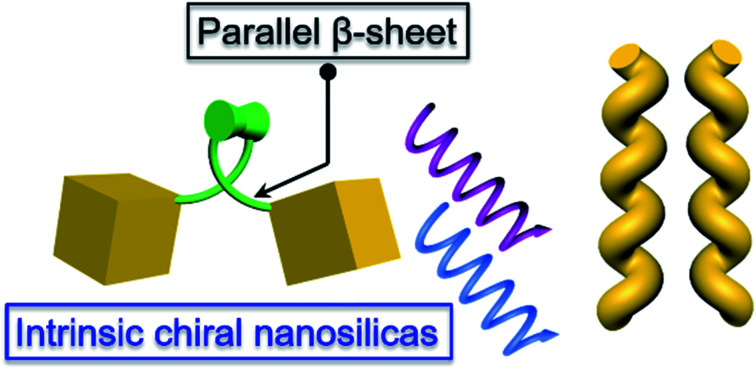

## Introduction

Natural organisms realize complicated functions and structures within chiral and helical environments, where noncovalent force-driven folding and hierarchical self-assembly play vital roles as a result of a long evolution process. The previous decade witnessed rapid development of helical and chiral macromolecular and supramolecular self-assembled systems across different levels, dimensions and sizes.^1,2^ Artificial helical systems are fabricated by the aggregation of small molecules, intramolecular folding or metal–ligand coordination of polymers, which not only mimic the helical structures from nature, but also exhibit abundant and diversified optical activities and interesting applications.^3^ Rational manipulation of the emergence, transfer, evolution, amplification and inversion of chirality deepens the understanding of the homochirality in nature and facilitates the fabrication of functional chiral materials.^4^ This intriguing topic has attracted considerable attention from multidisciplinary fields. However, there is still plenty of room and there are also several challenges in achieving precise control over molecular chirality and supramolecular chirality in helical systems from molecular to nanoscale levels. For instance, how do we push the helical packing of giant molecular nanoobjects with atomic precision?^5–9^

Molecular nanoparticles refer to atomically precise molecules with giant size over the nanometer scale. Metal clusters, such as polyoxometallates (POMs), proteins, fullerenes (C_60_) and polyhedral oligosiloxanes (POSS), are widely used molecular nanoparticles, which are shape and volume-persistent objects with size over 1 nm.^10^ The pronounced atomic precision and specific symmetry endow these nanoobjects with superiority compared to the self-assembled particles or synthetic metal particles with polydispersity. Considering the diversified size, dimension, surface functionalization and other chemicophysical properties, molecular nanoparticles have been used as building blocks in versatile self-assembly systems.^11–14^ Integration between chirality and molecular nanoparticles has sparked to enable fascinating helical systems with specific topology and functions.^15,16^ In this regard, tubulins self-assembled into helical microtubes;^17^ POMs and noble metal clusters could coassemble into helical structures within chiral templates;^14^ C_60_ encapsulated in helical polymers could achieve helical orientation.^8^ Among them, POSS are the smallest silica nanoparticles with diameter about 1 nm or higher depending on the grafted functional groups. POSS have been intensively incorporated into ordered architectures with versatile phases such as lamellar, bcc and a special A15 phase *via* delicate designs.^18–20^ Due to the high crystallinity and apolar nature, POSS have seldom been applied to construct helical systems, and a precise control over the helical orientation of POSS has not been well accomplished to date. Foldamers are defined as synthetic polymers or oligomers adopting a compact conformation.^21^ Typical examples include peptoids, oligonucleotides, oligoamides, aza-aromatic oligomers and tertiary aromatic units, which undergo folding into specific conformation *via* hydrogen bonds or aromatic interactions.^22^ Through intrinsic folding, helical structures with hierarchical levels of chirality are formed, providing a potential way to control the emergence and properties of helical POSS structures.

**Scheme 1:**
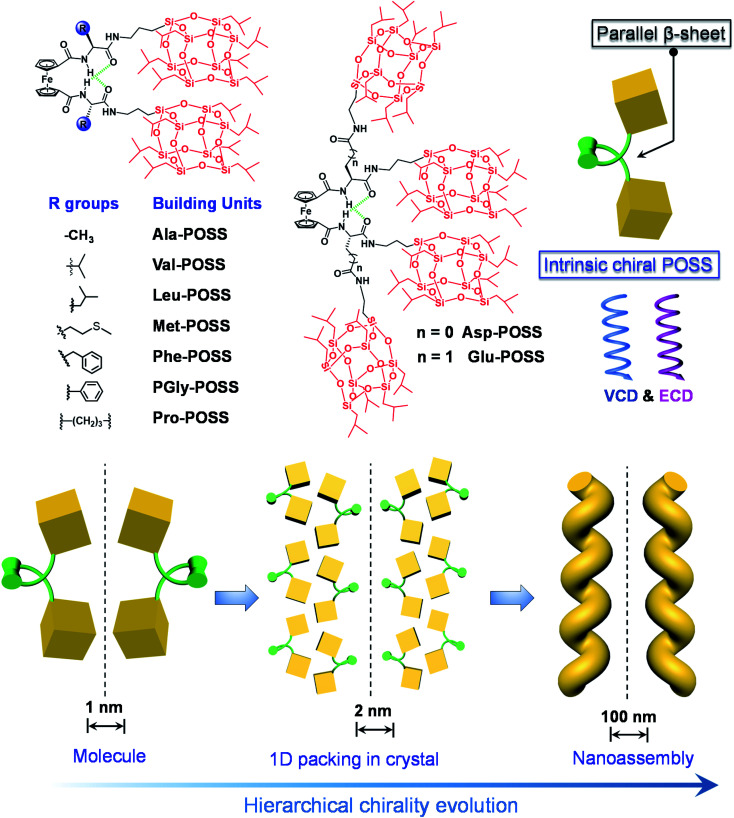
Molecular structures of ferrocenyl POSS conjugates as well as the schematic representation of the intrinsic chirality of POSS by intramolecular folding, and chirality evolution across hierarchical levels *via* self-assembly.

To realize the above goal, in this work, we designed an intramolecular foldable compound to precisely control and illustrate the screw sense and chirality within a folded molecule. To achieve this, we employed a 1,1′-ferrocenyl diamino acid core, which features intramolecular duplex hydrogen bonds between the amide and carboxylate domains, anchoring the diamino acid arms to afford a parallel β-sheet structure. The specific conformation, referred to as Herrick's conformation,^23,24^ could provide a feasible way to achieve chirality by varying the kinds and absolute configuration of amino acids. A small library of building units was built by conjugating isopropyl grafted POSS (T8 cage) to the 1,1′-ferrocenyl diamino acids. In the solid X-ray structures, intramolecular folding and the large size of POSS enabled the exclusive helical packing of POSS domains (Scheme 1). The folded chirality of POSS depended on the type of amino acids, which pronouncedly exhibited a helical arrangement in Glu-POSS with four POSS segments. This is the first illustration and precise determination of POSS folded helicity, which also showed abundant chiroptical responses including Cotton effects and vibrational circular dichroism signals. Furthermore, bottom-up self-assembly transmitted the intramolecular folded helicity in the microscale into nanohelical structures with identical homochirality to the crystal phase (Scheme 1). This work, on the design and fabrication of helical giant POSS particles using folded amino acids, paves a new way to precisely manipulate chirality and supramolecular chirality at the nanoscale, and is meaningful in the fabrication of novel organic chiroptical materials.

## Results and discussion

Due to the rotating ability of cyclopentadienyl planes, 1,1′-ferrocenyl diamino acids feature unique intramolecular hydrogen bonds between amides and carboxylic acids. The conformation, as proposed by Herrick *et al.*,^24^ was designated as the parallel β-sheet secondary structure,^25^ which anchors the diamino acid arms to introduce axial chirality from the point chirality of amino acids to the ferrocene group. Amino acids with diversified substituents showed intimate structure–property correlation with the chirality and chiroptical properties. In this work, iPr-POSS was conjugated to 1,1′-ferrocenyl diamino acids including Ala, Val, Leu, Met, Glu, Asp, Phe, PGly and Pro (here the abbreviations represent ferrocenyl diamino acids) *via* an amide condensation reaction. Nuclear magnetic resonance (NMR) and mass spectra indicated the successful synthesis (see details in the ESI†).

**Fig. 1:**
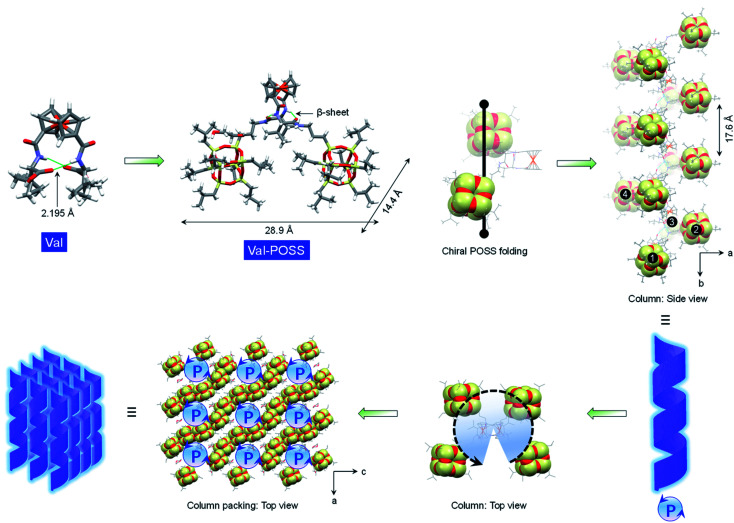
Geometry variation in the solid-state X-ray structures after conjugating the POSS segment to Val. Intramolecular folded POSS segments are highlighted in the CPK mode. In the packing mode, POSS segments grow into helices.

To probe the intramolecular folding motifs and intermolecular stacking of giant building units, crystals were cultivated by the liquid phase evaporation of dioxane/cyclohexane mixtures. Three crystal data including Val-POSS, Phe-POSS and Met-POSS were collected. In Val-POSS, two hydrogen bonds anchor the diamino acid arms between amides, where the amides adjacent to ferrocene contribute the hydrogen atoms (Fig. 1). The lengths of the hydrogen bonds were determined to be 2.099 and 2.345 Å, suggesting the biased orientation of POSS segments. Pristine Val without conjugated POSS exhibits intramolecular hydrogen bonds with a homo-length of 2.195 Å (CCDC 2056201). The conjugation of bulky POSS altered the orientation and distribution of substituents, which shall contribute to the spatial asymmetrical packing intramolecularly. Duplex hydrogen bonds support the Herrick conformation, whereby the dihedral angle between amino acid arms was determined to be 79.2°, slightly smaller than that of the pristine 1,1-ferrocene divaline with 83.2°. The dihedral angle results in nearly overlapped cyclopentadienyl planes. In the solid-state structure of Val-POSS, the modified POSS exhibits a cubical geometry with length around 10.0 Å (diagonal length = 14.4 Å). Consequently, the “V”-shaped Val-POSS has an overall nanosize structure, the lateral length of which is up to 28.9 Å. The nanosized POSS as well as the grafted isopropyl groups provide considerable structural persistence. Anchored by duplex hydrogen bonds, the two POSS segments exhibit specific spatial orientation. By fixing the ferrocenyl amino acid moieties, the two POSS “cubes” pack into a down-left-front/up-right-back orientation. While the front/back was determined by the absolute chirality of amino acids (l-type), the down-left/up-right affords chiral packing (Fig. 1). In the packing mode, Val-POSS features a *P*2_1_ space group. As a Sohncke group, *P*2_1_ is mathematically achiral disregarding the intrinsic shape of the segments.^26,27^ By considering the shape and geometry of building units, the *P*2_1_ space group affords chirality at hierarchical levels such as 2_1_ two-fold column or tilt chirality. Apart from the duplex intramolecular hydrogen bonds, the two amide groups were involved in the intermolecular hydrogen bonds to link the adjacent Val-POSS affording one-dimensional (1D) columns, where the POSS segments 1/2 and 3/4 from individual Val-POSS separately constitute a helical turn with a helical pitch of 17.6 Å (Fig. 1). In the depth cue representation, the orientation and extension of POSS generates a helical turn, which was designated as the *P*-handedness viewed along the *c* axis. In the top view, the helical columns are packed tetragonally. The solid X-ray structure verifies the vital role of hydrogen bonds that were formed intra- and intermolecularly, facilitating the formation of discrete and self-assembled helicates of POSS segments.

**Fig. 2:**
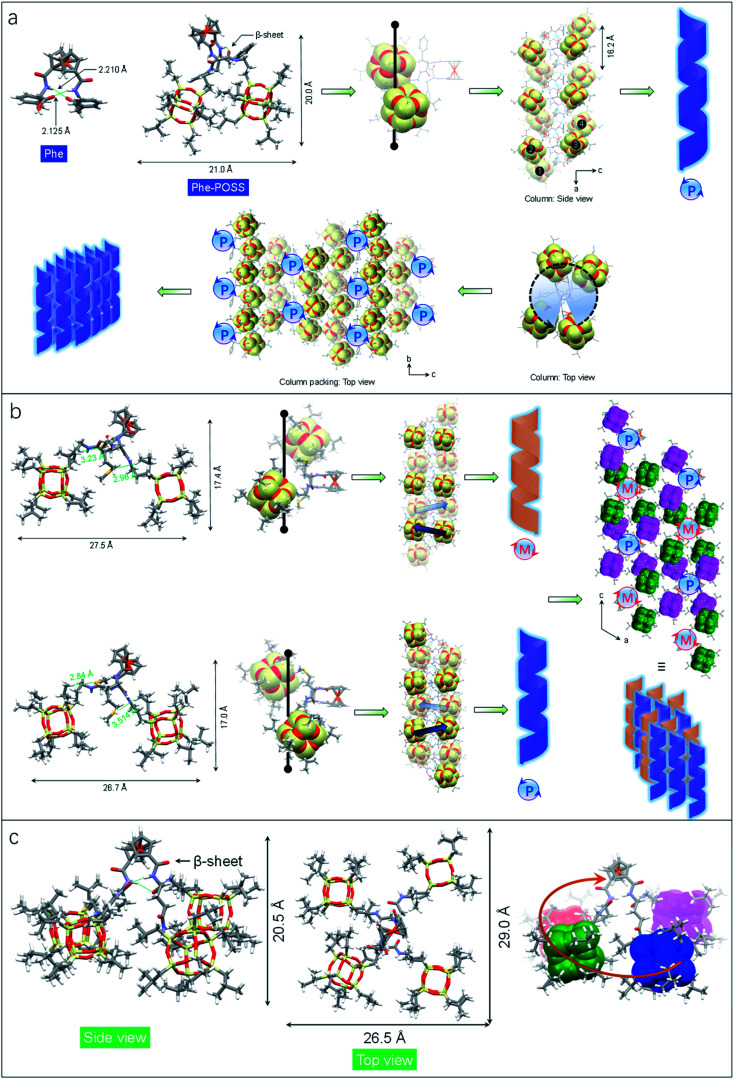
(a) Single crystal structure of Phe and Phe-POSS which features the helical folded conformation and packing. (b) Hetero-chiral structure of Met-POSS and the packing mode in the single crystal structure. The POSS segment was highlighted in CPK. (c) DFT optimized structure of Glu-POSS in different views.

The impact of amino acids on the folding and self-assembly behaviors was explored by the analysis of the solid-state structures of Phe-POSS and Met-POSS respectively (Fig. 2a and b). The pristine Phe with β-sheet and Herrick's conformation is supported by the typical duplex hydrogen bonds with lengths of 2.210 and 2.125 Å, which adapted to 2.179 and 2.438 Å respectively after conjugating with POSS segments (Fig. 2a). The relatively close packing between POSS segments results in an approximate 20 × 20 Å (length/height) size. The axial chirality of ferrocene is identical to that of Val-POSS, and it is also found that POSS “cubes” followed a different down-right-front/up-left-back orientation from that of Val-POSS. Phenylalanine and valine both possess substituents with aromatic and alkyl groups, which contributed to their differential conformational behaviors. In the packing mode, viewed along the *b* axis (side view), segments 1/2 and 3/4 from individual Phe-POSS constitute a staircase-like motif, which can be regarded as the tilt or 2_1_ columnar chirality, affording the *P*-handed helix. The handedness of 1D packing is independent of the intramolecular folding behavior. Viewed along the *a* axis, a hexagonal packing of the helices can be found.

Met-POSS adopted the identical β-sheet structure and Herrick's conformation, which enabled the formation of helical POSS folding as well (Fig. 2b). However, it is astonishing that the Met-POSS lattice contains two conformations, which share the same Herrick conformation in the ferrocenyl amino acid domains. The key difference between the conformations is in the orientation of POSS. The conformations exhibit an opposite helicity, resulting in the hetero-handed *M*- and *P*-helices respectively. In the packing mode, viewed along the *b* axis (top view), *P*- and *M*-columns pack adjacently into a supramolecular racemic phase. The major driving factors in the formation of the racemic mixture for POSS helices are associated with the existence of sulfur elements. Close contacts were found in both conformations between sulfur and methyl protons. The S⋯H interaction with distance around 3 Å hinders the close packing between POSS segments, resulting in a large lateral length up to 27.5 Å. It is believed that the homochirality originates from the transferred chirality from amino acids assisted by the duplex hydrogen bonds. Meanwhile, the bulky size of POSS moieties also contributes to the fixed chiral orientation. On account of the steric hindrance, the free rotation is forbidden even in the presence of alkyl spacers between the chiral centers and POSS segments. Thus, the specific chiral conformation is a result of the synergistic effect of hydrogen bonds, chiral centers and steric hindrance. The bulky space and relatively weak forces between POSS segments allow for swinging between the two geometries with opposite chiral orientations.

Compared to the above cases, Glu-POSS and Asp-POSS are special species conjugated by the four POSS segments. The conjugation of a large number of POSS with low polarity appended considerable steric hindrance with less polarity. Consequently, the crystallization of Glu-POSS and Asp-POSS is rather difficult. Density functional theory (DFT) optimization at the B3LYP/6-31g level of theory was applied to probe the geometry of Glu-POSS (Fig. 2c). Herrick's conformation and the β-sheet structure were maintained (length of hydrogen bonds: 1.835 and 2.269 Å), and the four bulk segments were distributed on the four corners. It displays a pyramid-like tetrahedral geometry, with length, width and height of 29.0, 26.5 and 20.5 Å respectively. According to the side view, an up-and-down orientation presents, on account of the uneven spacer lengths between POSS unit and ferrocenyl diamino acid core. From bottom to top, four POSS segments constitute a clockwise screw sense, suggesting that the β-sheet structure induces the formation of helical chirality intramolecularly. It is probably the first observation of helical nanoparticles within intramolecular folding, which may bring along an intriguing aggregation motif with chiroptical applications.

**Fig. 3:**
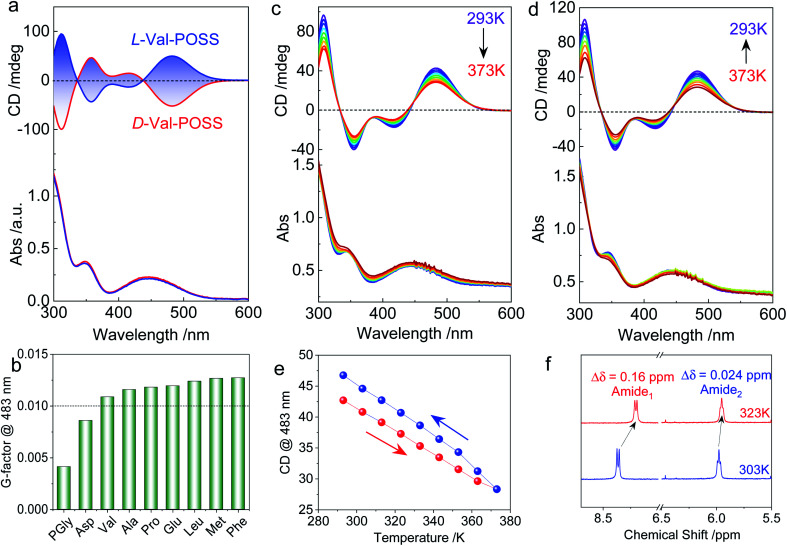
(a) CD spectra of Val-POSS in CHCl_3_ (1 mM) with different configurations. (b) Dissymmetry *g*-factors of different building units, “POSS” was omitted for clarity. (c–e) Temperature-variable CD spectra with heating and cooling processes of Val-POSS (1 mM in dioxane). (f) Partial ^1^H NMR spectra of Val-POSS in CDCl_3_ at different temperatures.

The above studies on X-ray structures represent chirality in the solid phase. Then we studied the chiroptical responses of various species in the solution phase. In the solution phase, all the nine building units exhibit well-resolved Cotton effects corresponding to the absorbance of the ferrocene unit (Fig. S21†), whose features are almost identical to the solid-state CD spectra (Fig. S22†). Thus, the consistency of CD signals in solution and the solid-state indicates the same chiral conformation. There is an exciton-type Cotton effect at the major absorbance around 450 nm, which is designated to the typical folded structure (electronic CD calculations using Phe without POSS segments as a model compound can be found in Fig. S23†). l- and d-Enantiomers afforded positive and negative maximum Cotton effects at 483 nm respectively, indicating the crucial effect of the absolute chirality of amino acids on the ferrocene (Fig. 3a). The type of amino acids has no impact on the Cotton effect, while the dissymmetry *g*-factors slightly vary in different species. Most building units gave rise to *g*-factors at around 0.01 (Fig. 3b), which are higher than that of most chiral chromophores.^27^ For the achiral chromophores directly conjugated to chiral moieties, the chromophores can be regarded as part of a large chiral entity, which lead to relatively small dot products through electric-dipole and magnetic-dipole transitions. In this situation, a relatively small CD signal would be generated with *g*-factors normally less than 10^−4^. In the present case, the large *g*-factor indicates the emergence of inherent chirality in the chromophore (ferrocenyl group). The folding by hydrogen bonds as well as the steric hindrance of POSS segments allows for a structure-persistent twisted ferrocene conformation with axial chirality, contributing to large *g*-factors.

As the intramolecular hydrogen bonds are vulnerable to temperature, temperature-variable CD spectroscopy was carried out (Fig. 3c and d). Heating the sample in dioxane from 293 K to 373 K witnessed the decreasing tendency of the Cotton effect with barely changed absorbance. After cooling down to room temperature, the Cotton effects recovered with a slightly enhanced CD signal (Fig. 3e). The irreversibility indicates that the thermal annealing process enhances the re-construction of hydrogen bonded folding. The destruction and re-construction of intramolecular hydrogen bonds contribute to the thermo-responsive chiroptical behavior. In the ^1^H NMR spectroscopy (Fig. 3f), raising the temperature from 303 to 323 K results in the upfield shift of amide protons. The chemical shift of Amide_1_ adjacent to the ferrocene core exhibited a pronounced shift value of 0.16 ppm, which is caused by the disassociation of intramolecular hydrogen bonds, in good agreement with the temperature-variable chiroptical activity. In contrast, Amide_2_, far away from ferrocene which was not involved in the intramolecular hydrogen bonding, exhibited low shift (Δ*δ* = 0.024 ppm).

Due to the structure-persistent property of the POSS-containing building units, the chiroptical spectra show negligible dependence on the solvent environments. Owing to the apolar nature of POSS, only few solvents with low polarity are considered good solvents, including CCl_4_, chloroform, dichloromethane (DCM), tetrahydrofuran (THF), dioxane, toluene, and methyl cyclohexane (MCH). Thus, polar solvents such as acetone, acetonitrile, methanol, dimethyl formamide, dimethyl sulfoxide, and water could hardly dissolve the samples. To exclude the solvent effect in chiroptical activities, the CD spectra of l-Val-POSS with 1 mM concentration in various solvents (Fig. S24†) were compared.^28–30^ CD spectra in various good solvents (*e.g.*, CCl_4_, chloroform, THF, dioxane, toluene and MCH) exhibit the same Cotton effect and similar intensity without any bathochromic or hyperchromic shift, which suggest that these solvents have a negligible effect on the chiroptical activities. The slightly changed CD intensity might be caused by the different absorption coefficients. In addition, temperature-variable CD spectra in CHCl_3_ were also obtained to verify the results with the above CD and NMR studies. As shown in Fig. S25,† increasing the temperature from 293 K to 333 K diminishes the Cotton effect, which would be recovered in the cooling process. The slight signal enhancement after the heating–cooling cycle is possibly contributed by the thermal annealing effect. Thus, the temperature-variable CD results in CHCl_3_ are in good agreement with those obtained using dioxane, further indicating the negligible solvent effect.

**Fig. 4:**
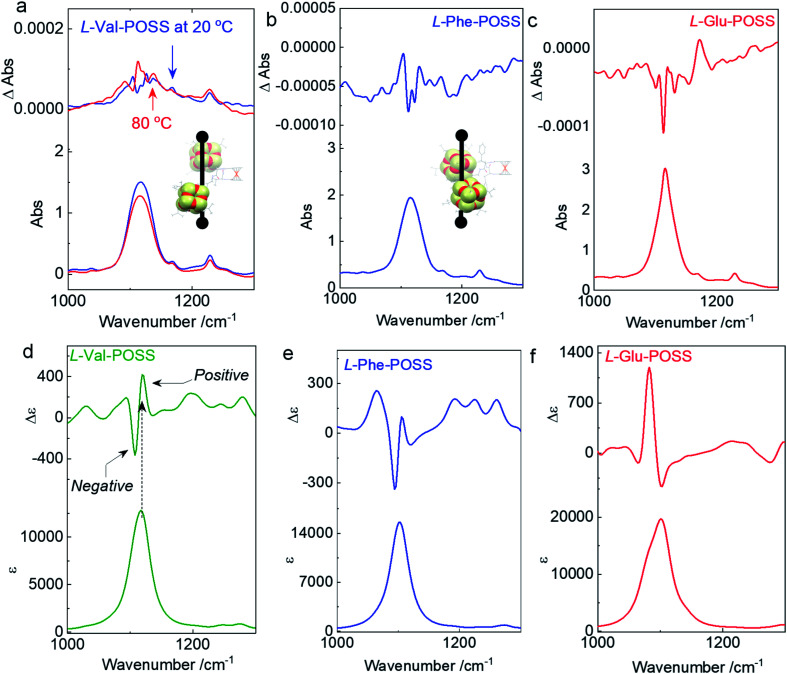
(a–c) VCD spectra of Val-POSS, Phe-POSS and Glu-POSS in CCl_4_ (50 mg mL^−1^) respectively. Insets display the corresponding intramolecular folding of POSS segments. (d–f) Calculated VCD spectra of Val-POSS, Phe-POSS and Glu-POSS respectively *via* time-dependent DFT (TDDFT) at B3LYP/6-31g(d) level of theory (full width at half maximum = 15 cm^−1^).

Single crystal, DFT computational results and ECD spectra show the chiral orientation of POSS segments *via* intramolecular folding. To verify the chiral packing in the solution phase, VCD was applied (Fig. 4 and S26–S29†) in CCl_4_ to exclude the pronounced solvent residue bands. The absorption of IR spectra at 1117 cm^−1^ is designated as the vibrational stretching band of Si–O bonds (Fig. S30†). The full FT-IR spectra as well as the partial regions on the amide-I and C=O stretching are displayed in Fig. S30.† In the solid state, there are multiple bands in the amide-I region for each sample. The stretching band at 3450 cm^−1^ corresponds to the free N–H group. The peak at around 3284 cm^−1^ is assigned as the hydrogen bonded N–H group.^31^ These observations suggest that partial N–H of amides is involved in the hydrogen bond formation, which are in good agreement with the single crystal structure profiles and the assumptions for intramolecular hydrogen bonded folding. Furthermore, the C=O stretching bands of each building unit appear from 1634 to 1628 cm^−1^. Such locations with low wavenumbers support the formation of the N–H⋯O=C hydrogen bond. In the solution state (CHCl_3_), the N–H⋯O=C hydrogen bond was also found (Fig. S31†), confirming the folding behavior in good solvents.

An apparent positive VCD signal was observed with intensity at 10^−4^ grade illustrated by delta absorbance (ΔAbs) at the specific region. In solution, the presence of an active VCD signal corresponds to the intrinsic chiral POSS segments. It is in good agreement with the single crystal profile. Although there are propyl spacers between POSS and amino acids with point chirality, chirality was transferred to POSS benefiting from the rigid Herrick conformation and β-sheet structure. Heating Val-POSS from room temperature to 80 °C slightly changed the VCD intensity, which however could hardly change the VCD sign and the major absorbance (Fig. 4a). Though the rising temperature would destroy the intramolecular hydrogen bonds, temperature-variable VCD indicates that POSS segments undergo bare structural relaxation at high temperatures, possibly aroused by the bulky size and the potential van der Waals interactions between isopropyl groups. Phe-POSS shows an opposite VCD sign at the absorbance of the Si–O bond (Fig. 4b). Thus, inversion in chirality is expected. According to the single crystal analysis, Val-POSS and Phe-POSS show distinct orientations (Fig. 1 and 2), which were well illustrated by the opposite VCD signs. Similarly, Glu-POSS shows a negative VCD sign as well (Fig. 4c), suggesting that the chiral packing of POSS shares the same propensity. Single crystal and optimized structures of these species were deposited for TDDFT studies to calculate VCD spectra. An exciton-type VCD signal was found in Val-POSS (Fig. 4d), and the Si–O band corresponds to the positive signal. In contrast, Phe-POSS and Glu-POSS display negative values, confirming the experimental results (Fig. 4e and f). Active C=O bands in VCD spectra can be found (Fig. S27†), which all feature a negative sign. The observation indicates that the chirality of C=O is determined by the absolute chirality of amino acids, in agreement with the axial chirality of the ferrocene unit. The calculated VCD spectra further confirm the retained chiral conformations in the solution phase as compared to the solid state. Despite the relatively weak signals, in contrast to that of CD spectra, different behavior was observed in VCD spectra. For the temperature-variable experiments, ^1^H NMR and CD spectra verified the disassociation of hydrogen bonds. However, the thermal resistance in VCD spectra shows that the chiral orientation between POSS segments may have less association with the hydrogen bonds, which may be due to the presence of flexible alkyl spacer chains.

**Fig. 5:**
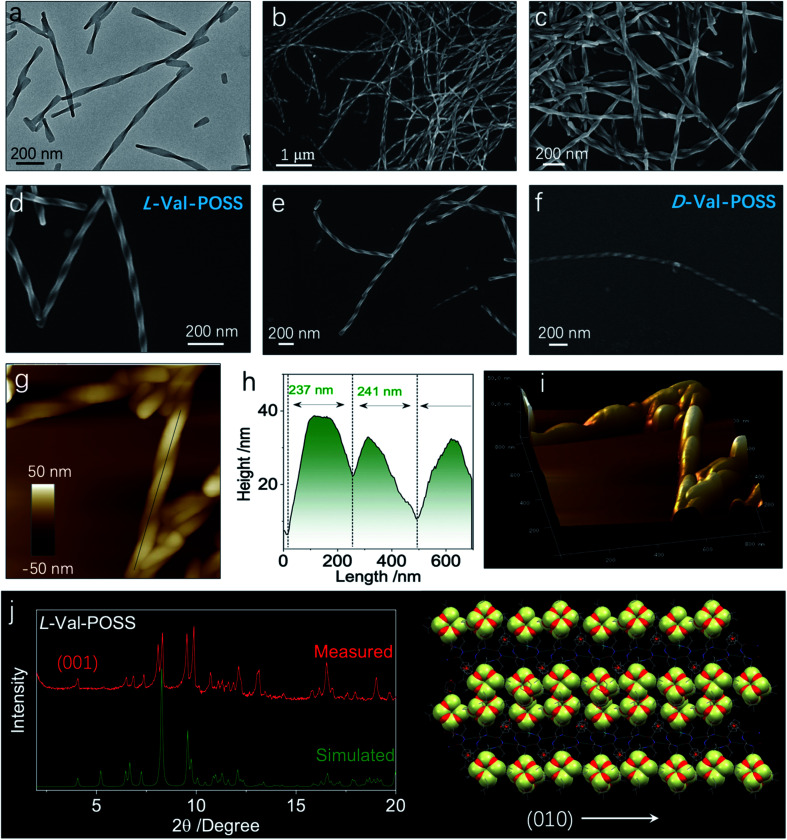
(a) TEM image of l-Val-POSS self-assembly (1 mM) in a THF/ACN mixture (1/9, v/v). (b–e) Corresponding SEM images of l-Val-POSS self-assembly at different magnifications. (f) SEM image of d-Val-POSS under the same conditions. (g and h) AFM (840 × 840 nm) and height profile of Val-POSS self-assembly. (i) Corresponding 3D AFM image. (j) Powder XRD pattern comparison as well as the speculated 1D growth of Val-POSS directed by hydrogen bonds.

Then, we wondered if the intramolecular folded chiral species could transmit the chirality to hierarchical levels, for example, supramolecular or nanoscale chirality. Bottom-up aggregation is a promising protocol. POSS with an Si–O bond and grafted alkyl chains is an apolar segment, which means that the building units with POSS could accomplish aggregation in polar solvents. Without POSS segments, Fc-amino acids cannot self-assemble in organic solvents, while in aqueous medium, Fc-amino acids self-assemble into vesicular nanoparticles without the emergence of helical structures.^32,33^ Interestingly, the appended POSS segments allow for the formation of well-defined helical structures in the THF/ACN mixture (1/9, v/v), contributed by the apolar nature of POSS, whereby the chirality evolution to a macroscopic scale is achieved. Through nanoprecipitation or solvent exchange method, in a tetrahydrofuran/acetonitrile (THF/ACN, 1/9, v/v) mixture, the building units (1 mM) underwent spontaneous aggregation into a colloidal dispersion as well as precipitate phases. Transmission electron microscopy (TEM) and scanning electron microscopy (SEM) were employed to inspect the morphologies. Val-POSS self-assembled into large-scale 1D fibrous structures (Fig. 5a–f). Although the fibers did not undergo infinite aggregation into an organogel phase, the length of the fibers was up to tens of micrometers with monodispersed width distribution. The monodispersity is also reflected in the macroscale chirality, whereby fibers show exclusive *P*-handedness. The supramolecular chirality is in good agreement with the 2_1_ column chirality found in the crystal phase, which suggests the successful transmission of chirality from the nanoscale scale to the micrometer scale across several orders of magnitude. The d-Configuration affords *M*-handedness (Fig. 5f). These structures show dependence on the absolute configuration of amino acids, whereby l- and d-type amino acids give *P*- and *M*-handedness, respectively. The helical fibers illustrate fine phase purity with respect to the supramolecular chirality. The helical pitch was determined to be around 240 nm as measured *via* the cross-section profile of atomic force microscopy (AFM). The aggregates were collected and deposited for the thin-film X-ray diffraction (XRD) study. Compared to the simulated powder pattern, the experimental XRD pattern shows similar peak locations with varied peak intensities (Fig. 5g). The pronounced appearance of the (001) plane indicates the perpendicular growth direction similar to the (010) plane which stands for the 2_1_ column helices supported by the hydrogen bonds.

For the Val-POSS, the helical self-assembly in the THF/ACN mixture shows a similar pattern to the simulated pattern from the single crystal, meaning that the molecular arrangement adopts a similar arrangement. In solution-processed bottom-up aggregation, 1D packing is preferred as compared to the thermodynamic growth conditions of the crystal. As predicted by molecular dynamic simulations, the 1D structures undergo twisting into chiral structures induced by the inherent chiral centers. Gazit *et al.*^34^ used molecular dynamic simulation and experimental methods to illustrate the helical structure formation in 1D crystalline aggregates, which are applicable to the present Val-POSS case wherein 1D molecular growth facilitates the helical structure formation. The high directionality of hydrogen bonds induces the 1D growth when the screw occurs by asymmetrical packing into nanohelices. On top of that, the self-assembly may give rise to chiral structures with similar molecular packing to the crystal phase. Nevertheless, the peak locations are similar, while the relative intensity of each plane is different (Fig. 5g). This difference is caused by the exposed planes in various phases, reflected in the intensity of each peak.

**Fig. 6:**
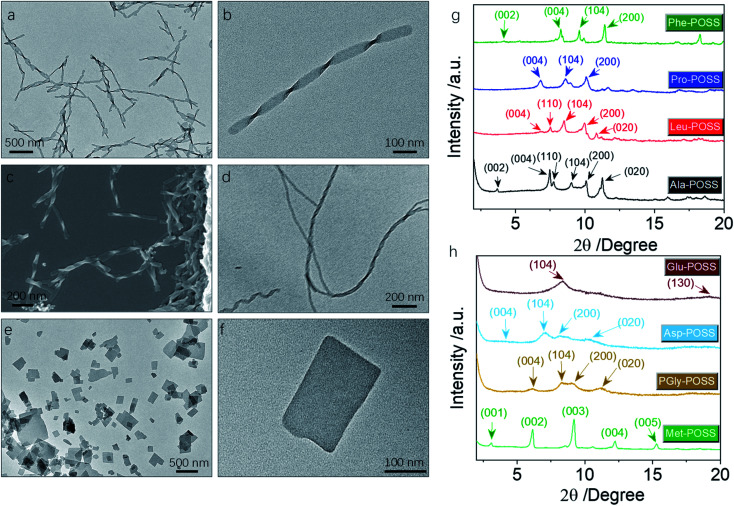
(a and b) TEM images of PGly-POSS (1 mM) self-assemblies in the THF/ACN mixture (1/9, v/v). (c) Corresponding SEM image of PGly-POSS (1 mM) self-assemblies. (d) TEM of Glu-POSS self-assembly under the same conditions. (e and f) TEM images of Pro-POSS plate assemblies under the same conditions. (g and h) XRD patterns and the plane assignment of different self-assemblies.

In addition to Val-POSS, other building units could self-assemble into nanohelical structures as well (Fig. 6a–d). Following the same self-assembly protocol, PGly-POSS afforded twisted architectures with length up to several micrometers (Fig. 6a–c). The helical pitch was measured to be 190 nm, less than that of Val-POSS. SEM image indicates the exclusive *P*-handedness, further verifying the determining role of amino acid in the supramolecular chirality in self-assemblies. The occurrence of supramolecular chirality was also found in the Glu-POSS self-assembly system, which gave a helical nanoarchitecture (Fig. 6d). The co-existence of single and double strand helical architectures implies an intertwining process that may have benefited from the abundant apolar POSS segments and enhanced inter-fiber interactions. Other than the three self-assembled helical aggregates, 6 other building blocks feature a crystallinity-induced self-assembly scenario (Fig. 6e, f, S32, and S33†). Crystalline 2D and 3D micro-objects were the dominant aggregates. For instance, Pro formed rectangular thin plates with lateral length about hundreds of nanometers (Fig. 6e and f). The 2D geometry is reminiscent of lamellar stacking. Such a situation also occurred in the case of Asp-POSS with nanoribbon architectures with low contrast, where layer-by-layer stacking is highly expected. The high crystallinity is attributed to the ferrocenyl and POSS segments that provide relatively strong interaction to induce growth along three dimensions. The crystalline property even hindered the kinetic aggregation method of nanoprecipitation, and consequently only some species accomplished chirality transfer across scales. The pioneering work by Cheng *et al.* evidenced the normal lamellar structure formation in POSS-containing building units without thermal annealing.^18^ In accordance with their observations, we also found that in the present chiral systems, lamellar structures are majorly adopted.

In powder XRD patterns, besides Val-POSS, Phe-POSS and Met-POSS show pronounced deviation of the simulated patterns from single crystal files, indicating that the solution-assembly adopted different ways of aggregation. It is interesting that the patterns of Ala-POSS, Leu-POSS, Pro-POSS and Phe-POSS possess 4–5 peaks ranging from 6 to 11 2theta degree, which could be assigned to the crystalline lamellar phase.^35^ In a few cases, they display the (002) plane with the missing (001) plane. The lamellar structure found in POSS-containing building units is attributed to the selected distribution of apolar POSS tails and polar ferrocenyl diamino acid heads, and within the individual lamellas also highly crystalline arrangement was found due to the existence of multiple planes. Met-POSS exclusively showed the entire lamellar peaks from (001) to (005) planes with a perfect distance ratio of 1 : 1/2 : 1/3 : 1/4 : 1/5, with a lattice parameter of 2.88 nm. However, other lamellar cases generated a rather loose stacking with a first order *d*-spacing value up to 5 nm, in good accordance with the previous POSS assemblies. PGly-POSS, Asp-POSS and Glu-POSS afforded hump peaks in contrast to other entities, probably contributed by the 1D aggregates without 3D molecular aggregation. These peaks can also be assigned to the lamellar stacking with retained planes and different lattice parameters. Powder XRD patterns illustrated the general formation of lamellar structures in ferrocene-diamino acid POSS building units, whereby chirality could be transmitted to hierarchical levels.

## Conclusions

In conclusion, we illustrated the design and fabrication of molecular chiral nanosilicas, chiroptical properties and self-assemblies. POSS segments were conjugated to the ferrocenyl diamino acids. The resultant Herrick conformation formation and folded parallel β-sheet structure enabled the asymmetric orientation of POSS segments disregarding the flexible spacers. The chirality of POSS was determined from the absolute chirality of amino acids with a pronounced substituent effect. The intrinsic chiral POSS molecules exhibited active VCD signals, which were persistent at high temperatures. Single crystal structures indicated the formation of helical motifs, which underwent aggregation into 1D helices with homo-chirality. This work reported the unprecedented formation of intrinsic helical silica nanoparticles with atomic precision in solid and solution states, creating new possibilities in the precise control of chirality and supramolecular chirality using molecular nanoparticles.

## Experimental

### Synthesis of ferrocene diamino acid methyl ester

The synthesis route is shown in Scheme S1.† 1,1′-Ferrocenedicarboxylic acid (600 mg) and l-valine methyl ester hydrochloride (1500 mg) were placed in a 250 mL flask. Then, hydroxybenzotriazole (HOBT, 200 mg), 4-dimethylaminopyridine (DMAP, 200 mg), 1-ethyl-3-(3-dimethylaminopropyl)carbodiimide (EDC, 1200 mg) and triethylamine (TEA, 1 mL) were added. The mixture was stirred in DCM (60 mL) at room temperature for 8 hours. When the reaction was complete, the mixture was poured into acidic water, extracted with DCM three times, and then reversely extracted twice, and finally purified using a silica gel column to obtain the pure product. Other compounds were synthesized using the same procedure.

### Synthesis of ferrocene diamino acids

The synthesis route is shown in Scheme S2.† The orange-yellow solid powder obtained above was dissolved in THF (15 mL), and then water (15 mL) was added under stirring. Finally, LiOH (600 mg) was added and stirred continuously for 30 min. When the reaction was complete, the reaction mixture was neutralized with hydrochloric acid, and subsequently extracted with DCM; an orange-yellow solid powder was obtained after rotary evaporation. Other compounds were synthesized using the same procedure.

### Synthesis of Val-POSS

The synthesis route is shown in Scheme S3.† Ferrocene divaline (500 mg) and POSS-NH_2_ (2.0 g) with HOBT (200 mg), DMAP (200 mg), EDC (1200 mg), and TEA (1 mL) were stirred in DCM (60 mL) at room temperature for 8 h. After the reaction was complete, the DCM solution was poured into acidic water, extracted with DCM and reversely extracted twice. The crude product was purified using a silica gel column and finally an orange-yellow powder was obtained by rotary evaporation. Other compounds were synthesized using the same procedure.

*Ala-POSS* was prepared as an orange-yellow solid in total 31% yield (659 mg, eluent: DCM/MeOH = 250 : 1). ^1^H NMR (400 MHz, CDCl_3_) *δ* 8.75 (s, 2H), 6.63 (s, 2H), 4.87 (s, 4H), 4.80–4.57 (m, 2H), 4.49 (s, 2H), 4.27 (s, 2H), 3.50 (s, 2H), 3.13 (s, 2H), 1.85 (dt, *J* = 11.0, 7.0 Hz, 14H), 1.65 (t, *J* = 8.2 Hz, 4H), 1.46 (s, 6H), 0.95 (d, *J* = 6.8 Hz, 84H), 0.60 (d, *J* = 7.0 Hz, 32H). ^13^C NMR (100 MHz, CDCl_3_) *δ* 174.91, 71.84, 71.04, 70.52, 70.05, 49.50, 42.28, 25.70, 25.68, 23.90, 23.85, 23.10, 22.50, 22.47, 17.41, 9.56. HRMS (TOF) *m*/*z* [M + H]^+^, calcd for C_80_H_159_FeN_4_O_28_Si_16_^+^, 2127.6793; found, 2127.5887.

*Val-POSS* was prepared as an orange-yellow solid in 32% yield (702 mg, eluent: DCM/MeOH = 250 : 1). ^1^H NMR (400 MHz, CDCl_3_) *δ* 8.36 (d, *J* = 8.0 Hz, 2H), 5.98 (t, *J* = 5.7 Hz, 2H), 4.84 (d, *J* = 2.3 Hz, 2H), 4.78–4.72 (m, 2H), 4.47 (d, *J* = 3.1 Hz, 2H), 4.29 (d, *J* = 2.6 Hz, 2H), 4.06–3.97 (m, 2H), 3.49 (dt, *J* = 13.6, 6.9 Hz, 2H), 3.19 (dd, *J* = 12.9, 6.1 Hz, 2H), 2.31–2.23 (m, 2H), 1.85 (dtd, *J* = 13.4, 6.7, 4.0 Hz, 14H), 1.66 (d, *J* = 8.8 Hz, 4H), 1.07 (d, *J* = 6.5 Hz, 6H), 0.95 (dd, *J* = 6.6, 1.7 Hz, 84H), 0.60 (d, *J* = 7.0 Hz, 32H). ^13^C NMR (100 MHz, CDCl_3_) *δ* 173.56, 71.92, 71.37, 69.90, 42.47, 28.94, 25.73, 25.72, 23.92, 23.87, 23.03, 22.49, 22.46, 20.24, 19.88, 9.73. HRMS (TOF) *m*/*z* [M + H]^+^, calcd for C_84_H_167_FeN_4_O_28_Si_16_^+^, 2183.7419; found, 2183.7245.

*d* *-Val-POSS* was prepared as an orange-yellow solid in 33% yield (730 mg, eluent: DCM/MeOH = 250 : 1). ^1^H NMR (400 MHz, CDCl_3_) *δ* 8.41 (d, *J* = 8.6 Hz, 2H), 6.23 (s, 2H), 4.88–4.79 (m, 2H), 4.77 (s, 2H), 4.47 (d, *J* = 2.9 Hz, 2H), 4.29 (d, *J* = 2.4 Hz, 2H), 4.05 (t, *J* = 8.7 Hz, 2H), 3.50 (dq, *J* = 13.6, 6.8 Hz, 2H), 3.18 (dt, *J* = 13.1, 6.4 Hz, 2H), 2.39–2.19 (m, 2H), 1.85 (dtd, *J* = 13.4, 6.7, 4.2 Hz, 14H), 1.67 (td, *J* = 10.2, 5.1 Hz, 4H), 1.07 (d, *J* = 6.3 Hz, 6H), 0.95 (dd, *J* = 6.7, 2.2 Hz, 84H), 0.60 (d, *J* = 7.1 Hz, 32H). ^13^C NMR (100 MHz, CDCl_3_) *δ* 173.57, 170.78, 76.17, 71.68, 71.16, 70.00, 69.83, 61.49, 42.36, 28.89, 25.70, 25.69, 23.90, 23.85, 23.01, 22.50, 22.47, 20.15, 19.81, 9.69. HRMS (TOF) *m*/*z* [M + H]^+^, calcd for C_84_H_167_FeN_4_O_28_Si_16_^+^, 2183.7419; found, 2183.7177.

*Leu-POSS* was prepared as an orange-yellow solid in 31% yield (700 mg, eluent: DCM/MeOH = 250 : 1). ^1^H NMR (400 MHz, CDCl_3_) *δ* 8.73 (s, 2H), 6.22 (s, 2H), 4.88 (s, 4H), 4.53 (d, *J* = 18.8 Hz, 4H), 4.28 (s, 2H), 3.45 (s, 2H), 3.16 (s, 2H), 1.91–1.80 (m, 20H), 1.65 (s, 4H), 0.95 (d, *J* = 6.6 Hz, 96H), 0.60 (d, *J* = 7.0 Hz, 32H). ^13^C NMR (100 MHz, CDCl_3_) *δ* 71.92, 71.13, 70.34, 70.15, 52.82, 42.36, 40.17, 25.74, 25.71, 24.87, 23.92, 23.87, 23.33, 23.10, 22.49, 22.48, 21.37, 9.65. HRMS (TOF) *m*/*z* [M + H]^+^, calcd for C_86_H_171_FeN_4_O_28_Si_16_^+^, 2211.7732; found, 2211.7567.

*Pro-POSS* was prepared as an orange-yellow solid in 30% yield (642 mg, eluent: DCM/MeOH = 250 : 1). ^1^H NMR (400 MHz, CDCl_3_) *δ* 9.09 (dd, *J* = 7.2, 4.1 Hz, 2H), 4.86 (s, 2H), 4.49 (d, *J* = 6.0 Hz, 4H), 4.28 (s, 4H), 3.73 (dt, *J* = 12.7, 7.0 Hz, 4H), 3.64 (dd, *J* = 12.1, 8.4 Hz, 2H), 3.22 (dd, *J* = 12.1, 7.0 Hz, 2H), 1.83 (ttd, *J* = 10.1, 6.4, 3.0 Hz, 26H), 0.93 (dd, *J* = 7.2, 5.7 Hz, 84H), 0.69–0.52 (m, 32H). ^13^C NMR (100 MHz, CDCl_3_) *δ* 171.00, 167.47, 78.14, 73.44, 70.17, 69.26, 67.26, 62.40, 46.22, 41.71, 31.15, 24.67, 22.85, 22.82, 21.88, 21.45, 21.41, 20.50, 8.68. HRMS (TOF) *m*/*z* [M + H]^+^, calcd for C_84_H_163_FeN_4_O_28_Si_16_^+^, 2179.7106; found, 2179.7111.

*Met-POSS* was prepared as an orange-yellow solid in 31% yield (730 mg, eluent: DCM/MeOH = 250 : 1). ^1^H NMR (400 MHz, CDCl_3_) *δ* 8.71 (s, 2H), 6.44–6.20 (m, 2H), 4.89 (s, 2H), 4.79 (s, 2H), 4.69 (s, 2H), 4.52 (s, 2H), 4.30 (s, 2H), 3.40 (s, 2H), 3.23 (s, 2H), 2.63 (d, *J* = 22.9 Hz, 4H), 2.18 (s, 2H), 2.09 (s, 6H), 1.96 (s, 2H), 1.90–1.81 (m, 14H), 1.66 (s, 4H), 0.95 (dd, *J* = 6.6, 1.6 Hz, 84H), 0.60 (dt, *J* = 7.1, 2.6 Hz, 32H). ^13^C NMR (100 MHz, CDCl_3_) *δ* 172.81, 169.97, 74.41, 71.01, 70.36, 69.20, 52.20, 41.44, 29.99, 29.72, 24.71, 24.69, 24.66, 22.88, 22.83, 22.82, 21.91, 21.44, 14.47, 8.62. HRMS (TOF) *m*/*z* [M + H]^+^, calcd for C_84_H_167_FeN_4_O_28_S_2_Si_16_^+^, 2247.6860; found, 2247.6077.

*Phe-POSS* was prepared as a light yellow solid in 30% yield (680 mg, eluent: DCM/MeOH = 250 : 1). ^1^H NMR (400 MHz, CDCl_3_) *δ* 8.81 (s, 2H), 7.37–7.18 (m, 10H), 5.93 (s, 2H), 4.79 (t, *J* = 24.6 Hz, 6H), 4.49 (s, 2H), 4.29 (s, 2H), 3.25 (t, *J* = 44.0 Hz, 8H), 1.91–1.77 (m, 14H), 1.46 (s, 4H), 1.02–0.87 (m, 84H), 0.59 (t, *J* = 7.2 Hz, 32H). ^13^C NMR (100 MHz, CDCl_3_) *δ* 172.37, 169.67, 136.60, 128.28, 127.67, 125.86, 74.75, 70.88, 70.26, 69.12, 55.62, 41.47, 36.47, 24.69, 24.66, 22.86, 22.83, 21.70, 21.48, 21.43, 8.52. HRMS (TOF) *m*/*z* [M + H]^+^, calcd for C_92_H_167_FeN_4_O_28_Si_16_^+^, 2279.7419; found, 2279.7478.

*PGly-POSS* was prepared as an orange-yellow solid in 32% yield (720 mg, eluent: DCM/MeOH = 250 : 1). ^1^H NMR (400 MHz, CDCl_3_) *δ* 8.68 (s, 2H), 7.40 (d, *J* = 62.3 Hz, 10H), 5.97 (s, 2H), 5.74 (s, 2H), 4.87 (s, 4H), 4.51 (s, 2H), 4.33 (s, 2H), 3.20 (s, 2H), 3.02 (s, 2H), 1.90–1.79 (m, 14H), 1.53 (s, 4H), 0.98–0.89 (m, 84H), 0.59 (t, *J* = 6.7 Hz, 32H). ^13^C NMR (100 MHz, CDCl_3_) *δ* 170.13, 168.98, 136.73, 127.83, 127.45, 127.18, 75.16, 70.96, 70.63, 70.46, 68.91, 55.79, 41.51, 24.68, 24.66, 22.85, 22.82, 21.75, 21.43, 21.39, 8.41. HRMS (TOF) *m*/*z* [M + H]^+^, calcd for C_90_H_163_FeN_4_O_28_Si_16_^+^, 2251.7106; found, 2251.7398.

*Asp-POSS* was prepared as a light yellow solid in 25% yield (980 mg, eluent: DCM/MeOH = 250 : 1). ^1^H NMR (400 MHz, CDCl_3_) *δ* 8.08 (d, *J* = 7.8 Hz, 2H), 6.68 (s, 2H), 4.85 (d, *J* = 10.4 Hz, 4H), 4.52 (d, *J* = 31.8 Hz, 4H), 4.32 (s, 2H), 3.39–3.08 (m, 8H), 2.83 (s, 2H), 2.62 (s, 2H), 1.85 (dq, *J* = 13.3, 6.6 Hz, 28H), 1.56 (d, *J* = 8.2 Hz, 8H), 1.06–0.81 (m, 168H), 0.59 (p, *J* = 7.4, 6.6 Hz, 64H). ^13^C NMR (100 MHz, CDCl_3_) *δ* 173.93, 170.62, 70.80, 51.77, 43.43, 43.16, 26.48, 26.46, 24.66, 24.63, 24.61, 23.62, 23.47, 23.26, 23.21, 23.16, 10.42. HRMS (TOF) *m*/*z* [M + H]^+^, calcd for C_144_H_297_FeN_6_O_54_Si_32_^+^, 3926.2639; found, 3926.2296.

*Glu-POSS* was prepared as a yellow solid in 26% yield (985 mg, eluent: DCM/MeOH = 250 : 1). ^1^H NMR (400 MHz, CDCl_3_) *δ* 8.63–8.42 (m, 2H), 6.97–6.69 (m, 2H), 5.84 (s, 2H), 4.83 (d, *J* = 7.7 Hz, 4H), 4.52 (d, *J* = 26.0 Hz, 4H), 4.30 (s, 2H), 3.37–3.10 (m, 8H), 2.39 (s, 4H), 2.17 (s, 2H), 1.99 (dd, *J* = 14.6, 7.4 Hz, 2H), 1.84 (dqt, *J* = 12.9, 8.6, 4.5 Hz, 28H), 1.69–1.50 (m, 8H), 1.03–0.85 (m, 168H), 0.60 (td, *J* = 6.8, 6.2, 2.9 Hz, 64H). ^13^C NMR (100 MHz, CDCl_3_) *δ* 174.38, 173.20, 171.55, 78.12, 71.97, 33.43, 53.69, 43.28, 33.43, 26.50, 26.47, 24.66, 24.61, 23.64, 23.27, 23.24, 23.21, 10.45, 10.28. HRMS (TOF) *m*/*z* [M + H]^+^, calcd for C_146_H_301_FeN_6_O_54_Si_32_^+^, 3954.2952; found, 3954.2988.

## Data availability

The data that support the findings of this work are included in the ESI.†

## Author contributions

P. Xing and Y. Zhao conceived the research idea. Z. Zong carried out the experiments and collected the data. A. Hao participated in the analysis and discussions. All authors contributed to the final manuscript.

## Conflicts of interest

There are no conflicts to declare.

## Supplementary Material

SC-013-D2SC00793B-s001

SC-013-D2SC00793B-s002
