# Supplementary material for: Chiral molecular nanosilicas
Source: Chem Sci. 2022 Mar 14;13(14):4029–40. doi: 10.1039/d2sc00793b (PMC8985511; doi:10.1039/d2sc00793b)
Supplement: SC-013-D2SC00793B-s001 [file SC-013-D2SC00793B-s001.pdf]

Electronic Supplementary Material (ESI) for Chemical Science.
This journal is © The Royal Society of Chemistry 2022

# Electronic Supplementary Information

## Chiral Molecular Nanosilicas

Zhaohui Zong,[a] Aiyou Hao,[a] Pengyao Xing*,[a,b] and Yanli Zhao*[b]

[a]*Key Laboratory of Colloid and Interface Chemistry of Ministry of Education and School of Chemistry and Chemical Engineering, Shandong University, Jinan 250100, People's Republic of China.*

[b]*Division of Chemistry and Biological Chemistry, School of Physical and Mathematical Sciences, Nanyang Technological University, 21 Nanyang Link, Singapore 637371, Singapore.*

*Email: xingpengyao@sdu.edu.cn; zhaoyanli@ntu.edu.sg*

**Experimental section**

**Materials**

1,1'-Ferrocnedocarboxylic acid and all amino acid methyl ester hydrochlorides were purchased from HEOWNS Biochemical Technology Co., LTD, China. POSS-$NH_2$ was purchased from Xi'an Qiyue Biological Technology Co., Ltd. All reagents were used without further purification in this work. *L*-Configuration amino acids are used throughout this supporting information unless otherwise specified.

**Characterizations**

$^1H$ NMR spectra, $^{13}C$ NMR spectra and temperature-variable $^1H$ NMR spectra were collected by BRUKER AVANCE III HD 400. High-Resolution Mass Spectra (HR-MS) were performed on an Agilent Q-TOF 6510. Circular dichroism (CD) spectra, temperature-variable CD spectra and solid CD spectra were measured with an Applied Photophysics ChirascanV100 model. Vibrational circular dichroism (VCD) spectra were obtained by a JASCO FVS-6000. VCD testing parameters: data interval: 1.92847 $cm^{-1}$; IR accumulation: 16; VCD accumulation: 6000;

resolution: 8 $cm^{-1}$; scanning speed: 4 mm/sec; polarizer angle: -45°; cell length: 50 μm. Transmission electron microscope (TEM) images were measured on a HITACHI HT-7700. The samples for TEM detection were dropped in the copper grid and air-dried. Scanning Electron Microscopy (SEM) images were obtained by Carl Zeiss G300 FE-SEM System. The samples for SEM detection were dropped on the silicon wafers and air-dried. Atomic force microscopy (AFM) testing was conducted with a Bruker Bioscope Resolve and operated in tapping mode at ambient temperature. The AFM samples were dropped on the mica wafer and dried. Single crystal data were collected on a Rigaku XtaLAB Synergy. Powder X-ray diffraction (XRD) patterns were collected on a German Bruker/D8 Advanced diffractometer with Cu Kα radiation (λ = 0.15406 nm, voltage 40 KV, current 40 mA). The samples for XRD were casted onto cover glasses (18 mm × 18 mm) and dried to form thin films.

**Solid-state CD spectra**

To obtain solid samples, building units were dissolved in $CH_2Cl_2$, followed by air-drying. The solid samples were then mixed in a mortar with KBr (5 mg solid sample in 200 mg KBr) under an infrared lamp. The ground mixtures were tableted into transparent tablets and tested.

**Computational details**

The geometric conformations of the molecules (Val-POSS, Met-POSS, Phe-POSS) used in the calculations were obtained from the corresponding single crystals. Then optimization to the energy-minimum structure in the gas phase was performed using Gaussian16 software with B3LYP/6-31g level of theory. Based on the optimized geometry, vibrational circular dichroism (VCD) spectra and electronic circular dichroism (ECD) spectra were calculated to obtain with basic set of B3LYP/6-311g. The geometric conformation of Glu-POSS was built in Gaussian View 06 and then optimized to the energy-minimum structure using Gaussian 16 software. The basis set used in the optimization process was B3LYP/6-31g. Vibrational circular dichroism (VCD) spectrum and electronic circular dichroism (ECD) spectrum were calculated based on the above pre-settings.

In the optimization process, the convergence criteria of optimization were as follows: Maximum Force 0.000015, RMS Force 0.000010, Maximum Displacement 0.000060, RMS

Displacement 0.000040. To ensure that the optimized geometry was at a minimum, all geometry optimizations were followed by a frequency calculation and only positive frequencies were obtained.

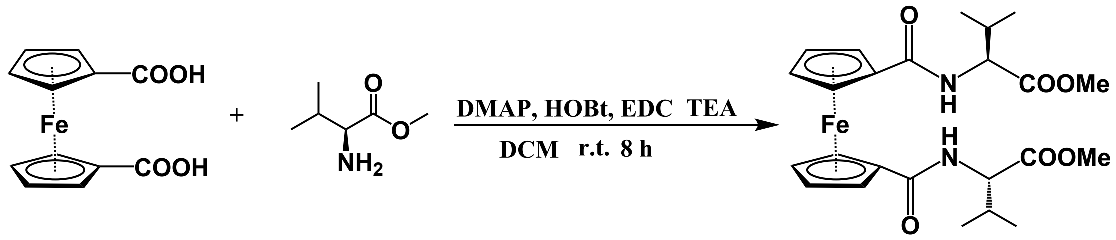


**Scheme S1**. Synthesis route of ferrocene diamino acid methyl ester.

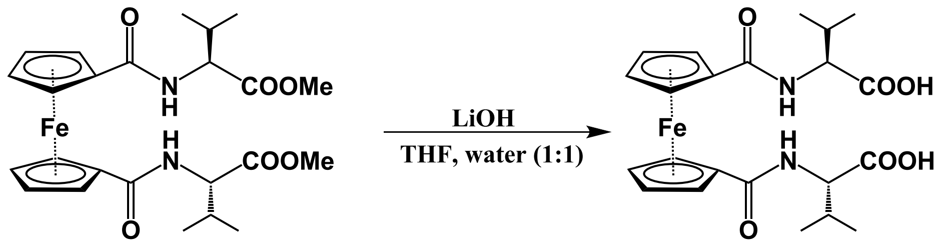


**Scheme S2**. Synthesis route of ferrocene diamino acid.

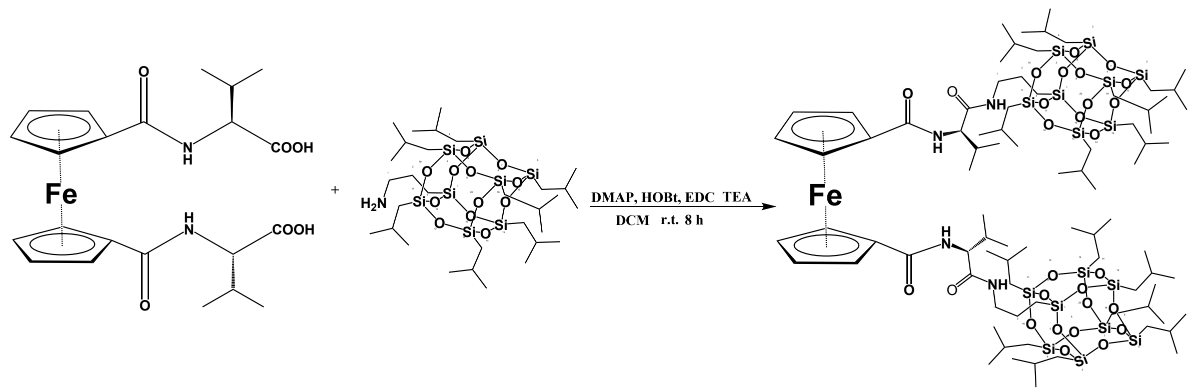


**Scheme S3**. Synthesis route of Val-POSS.

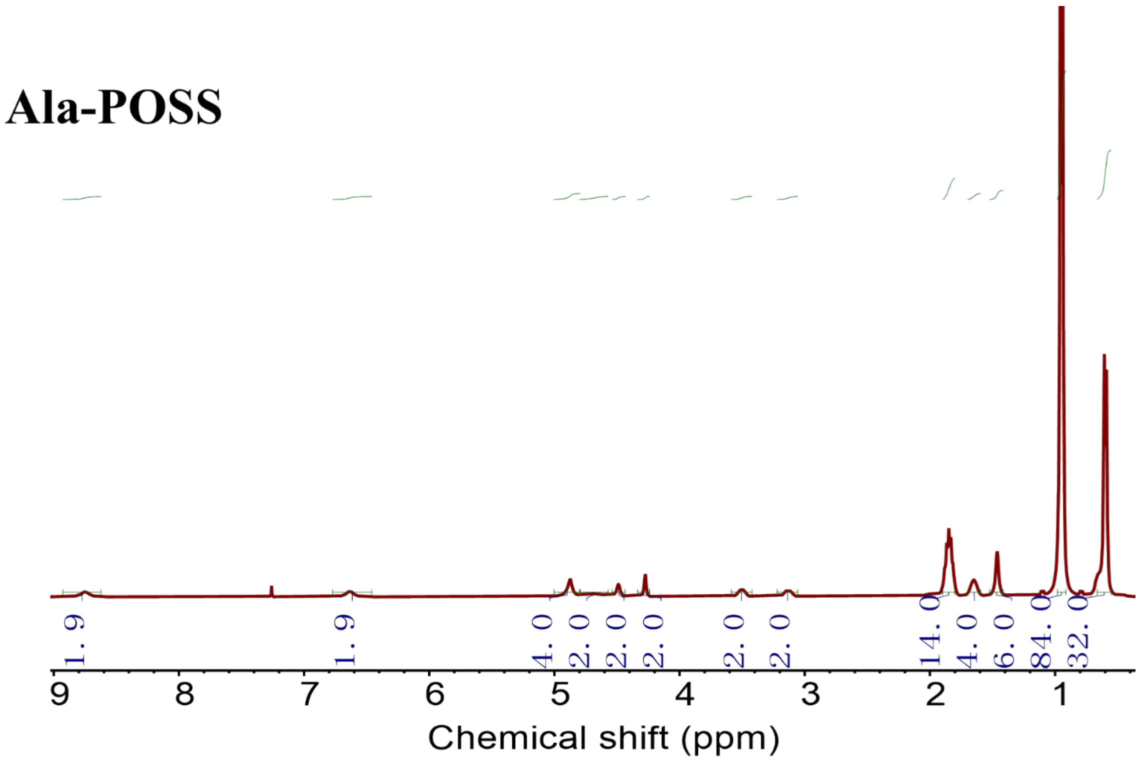


**Figure S1**. ¹H NMR spectrum of Ala-POSS.

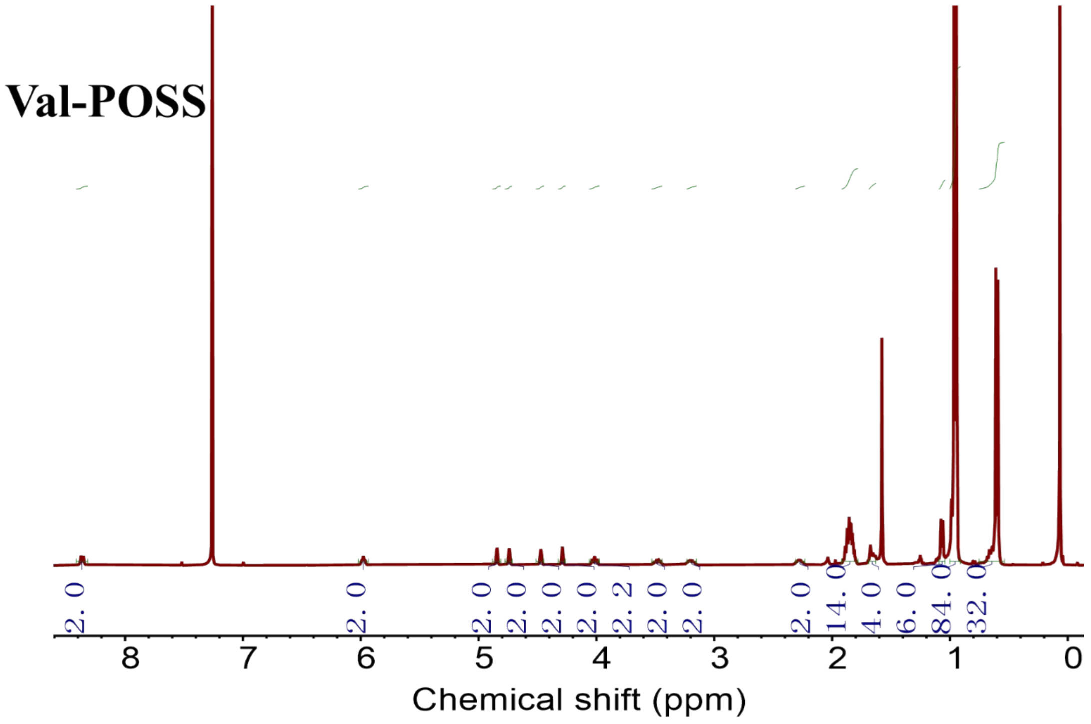


**Figure S2**. ¹H NMR spectrum of Val-POSS.

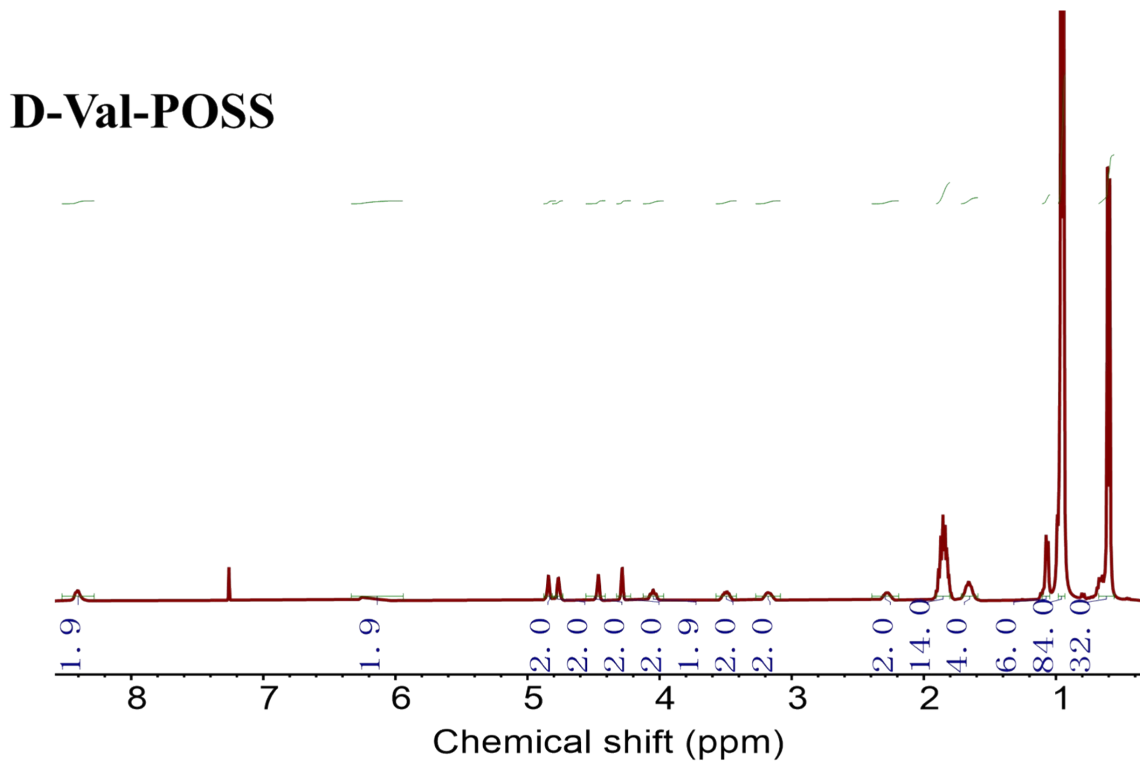


**Figure S3**. $^{1}$H NMR spectrum of D-Val-POSS.

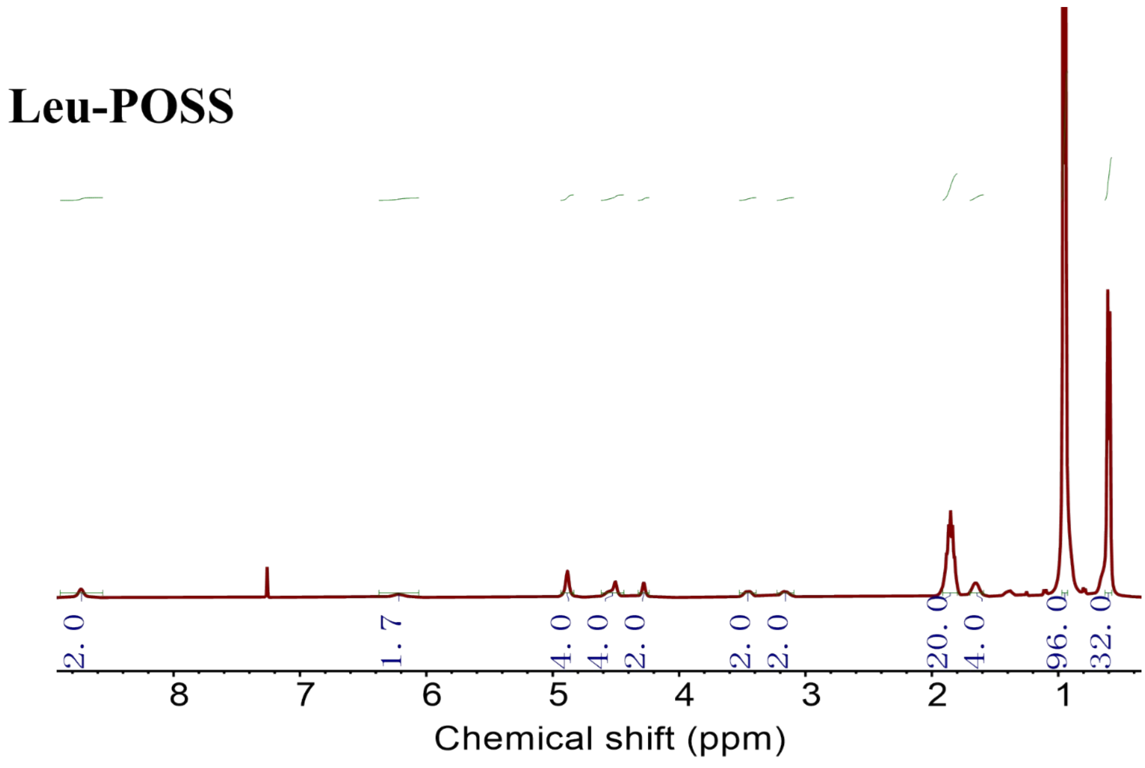


**Figure S4**. $^{1}$H NMR spectrum of Leu-POSS.

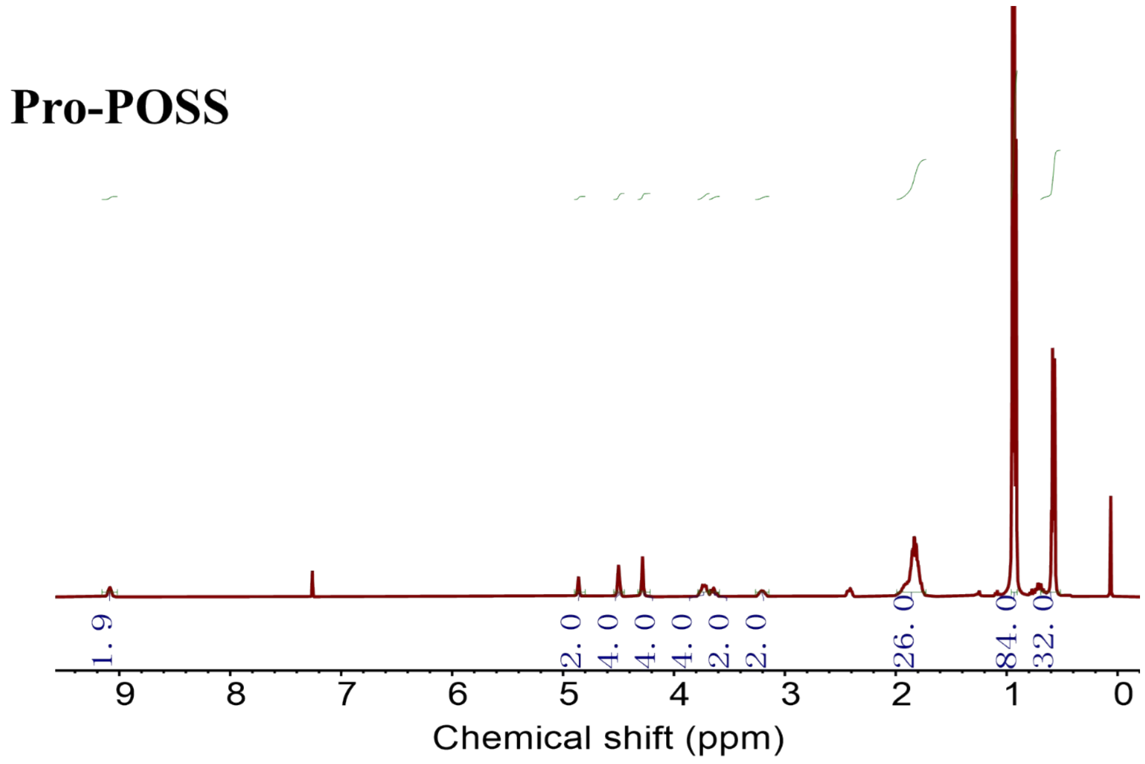


**Figure S5**. $^1$H NMR spectrum of Pro-POSS.

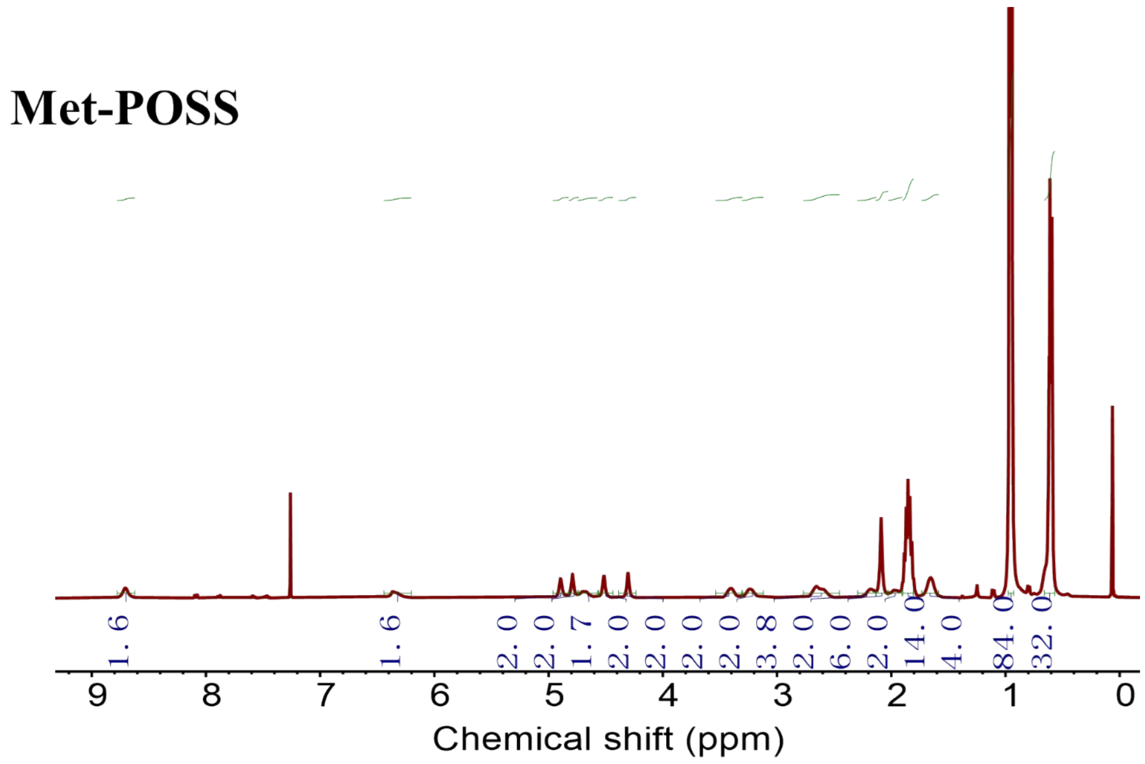


**Figure S6**. $^1$H NMR spectrum of Met-POSS.

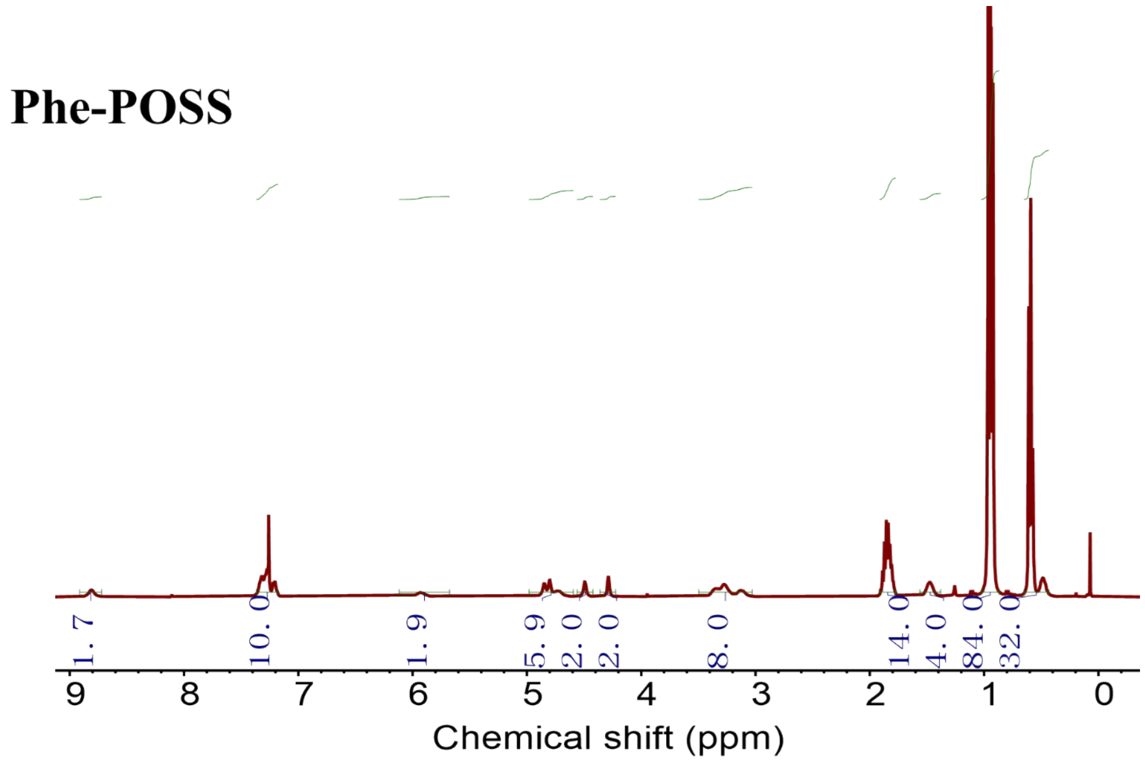


**Figure S7**. $^{1}$H NMR spectrum of Phe-POSS.

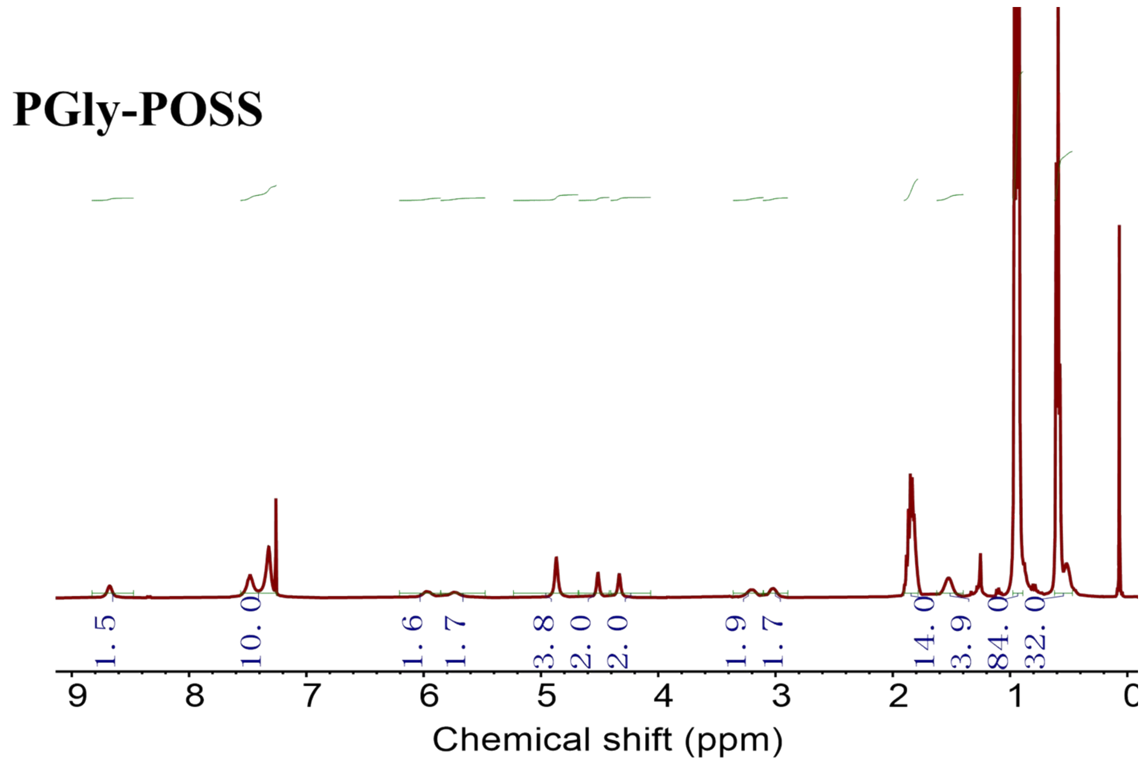


**Figure S8**. $^{1}$H NMR spectrum of PGly-POSS.

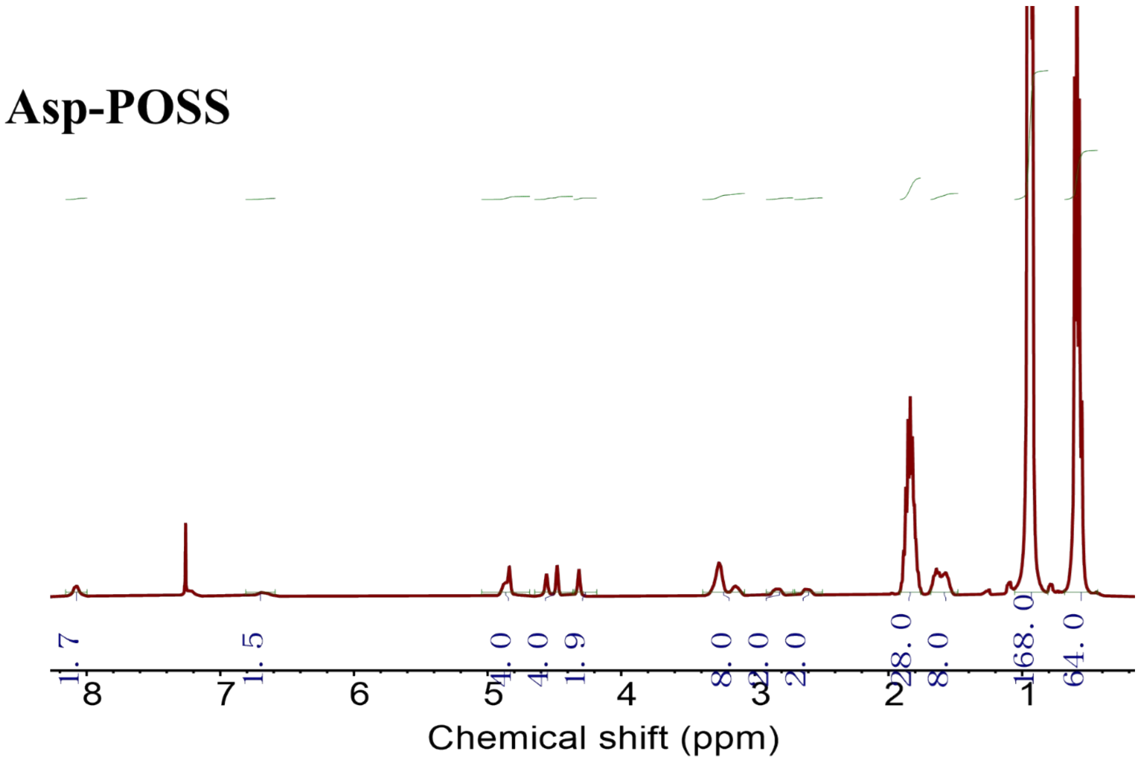


**Figure S9**. $^{1}H$ NMR spectrum of Asp-POSS.

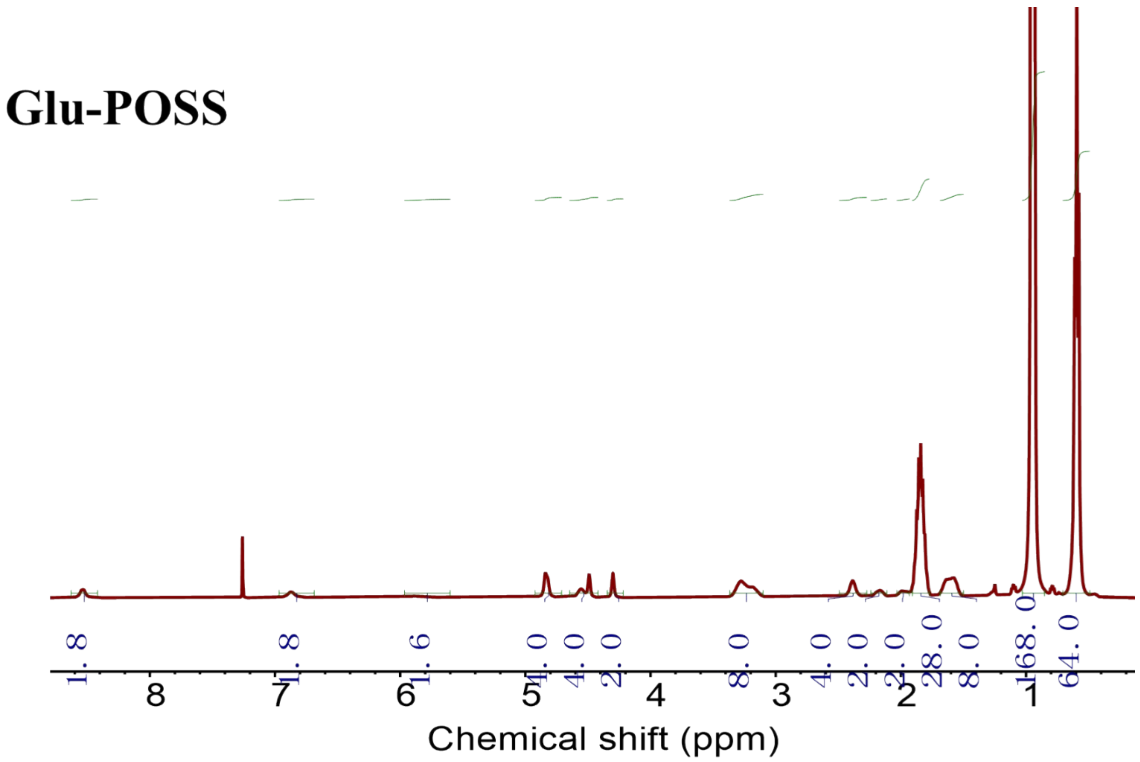


**Figure S10**. $^{1}H$ NMR spectrum of Glu-POSS.

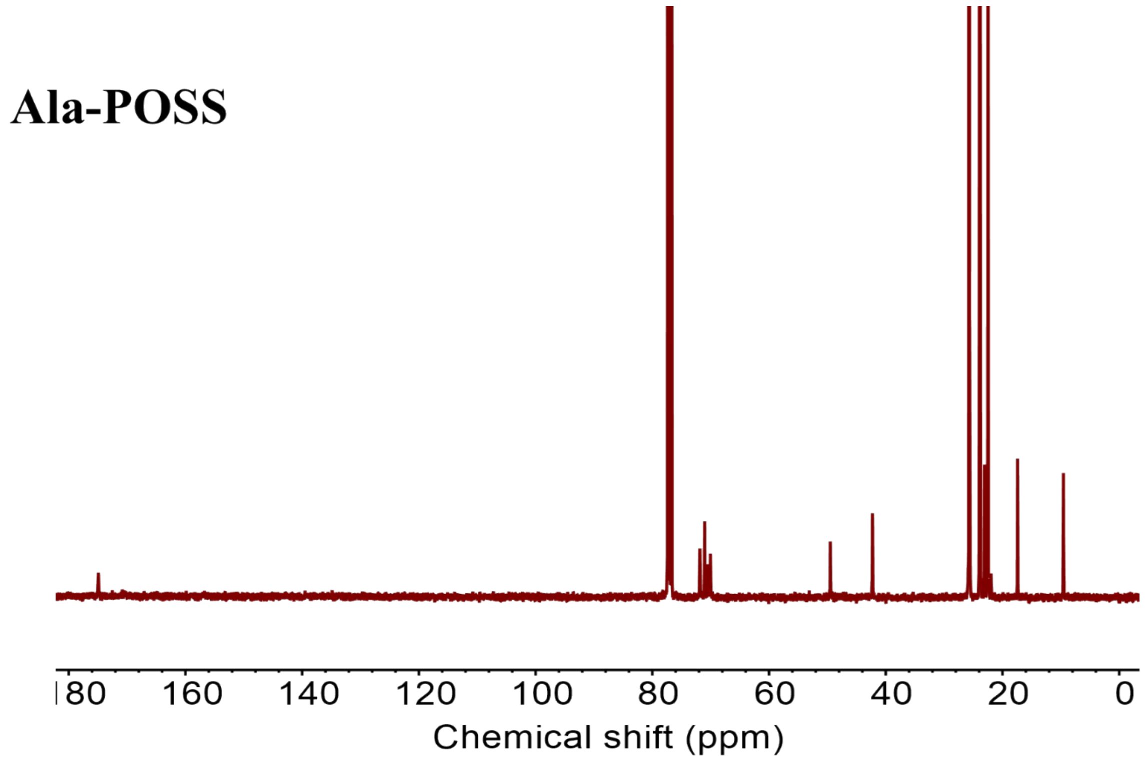


**Figure S11**. $^{13}C$ NMR spectrum of Ala-POSS.

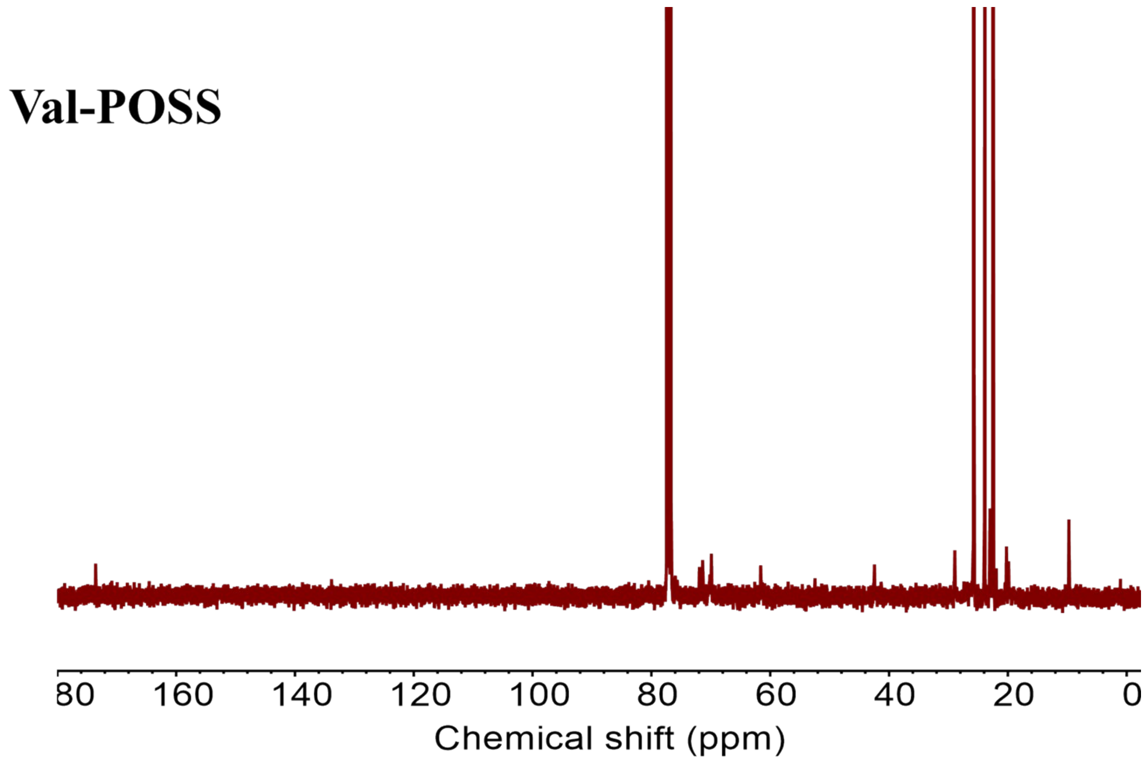


**Figure S12**. $^{13}C$ NMR spectrum of Val-POSS.

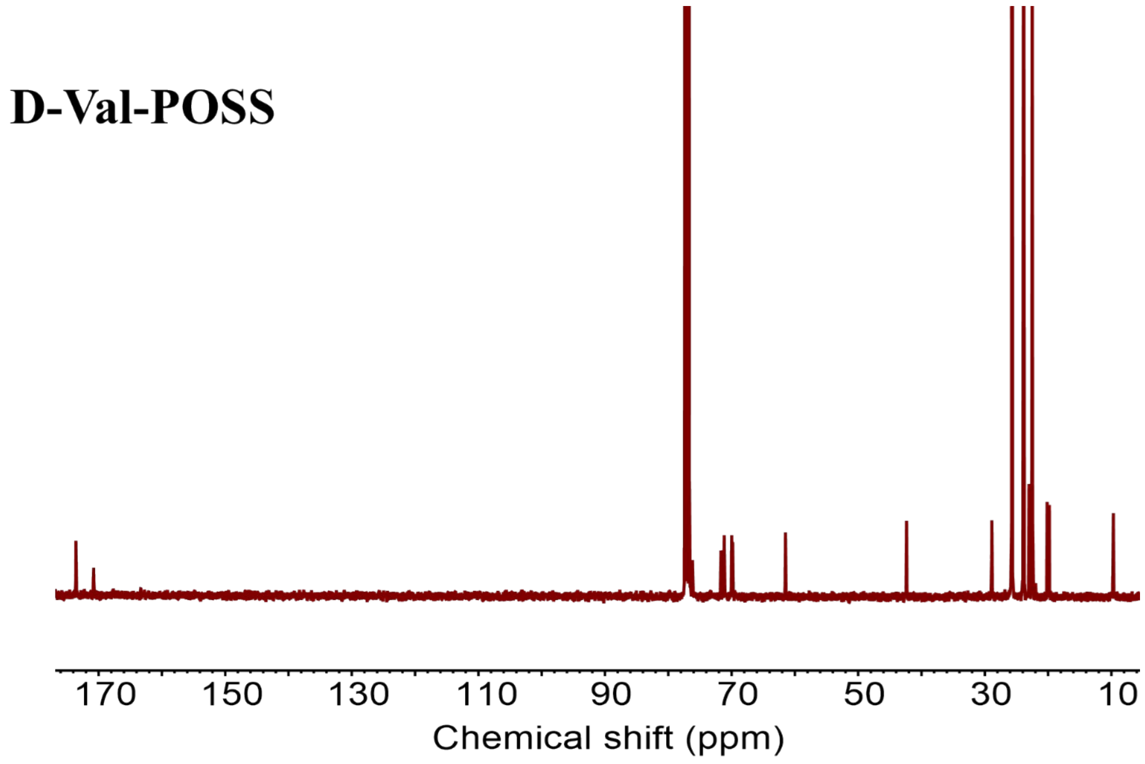


**Figure S13**. $^{13}$C NMR spectrum of D-Val-POSS.

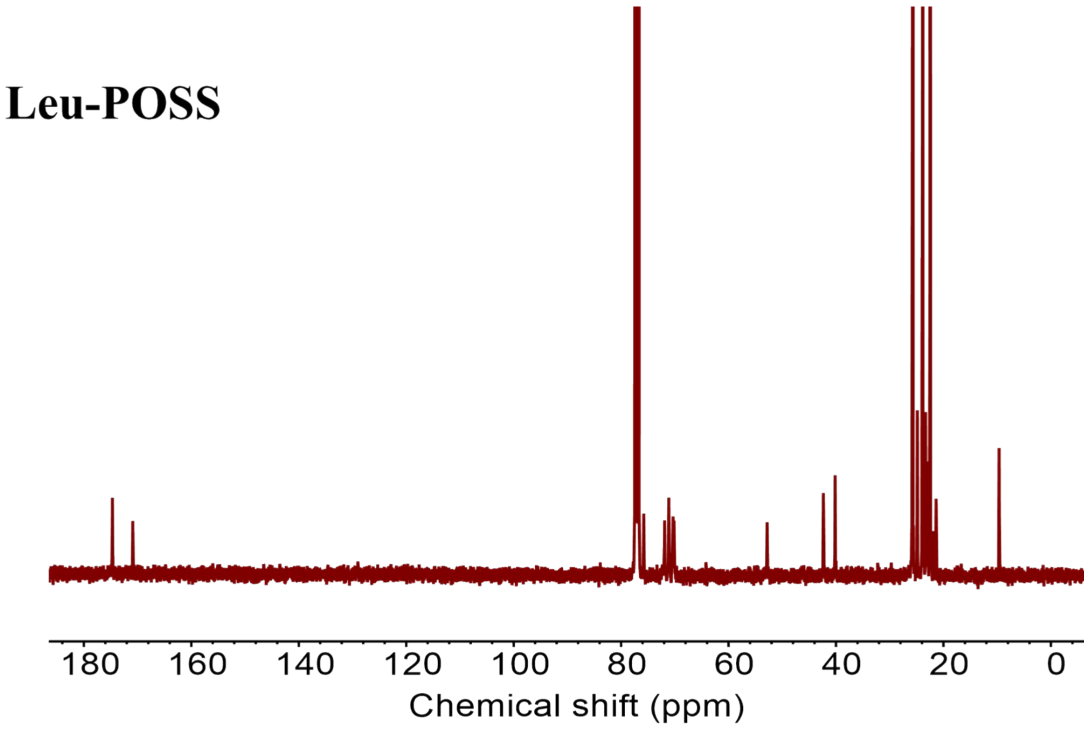


**Figure S14**. $^{13}$C NMR spectrum of Leu-POSS.

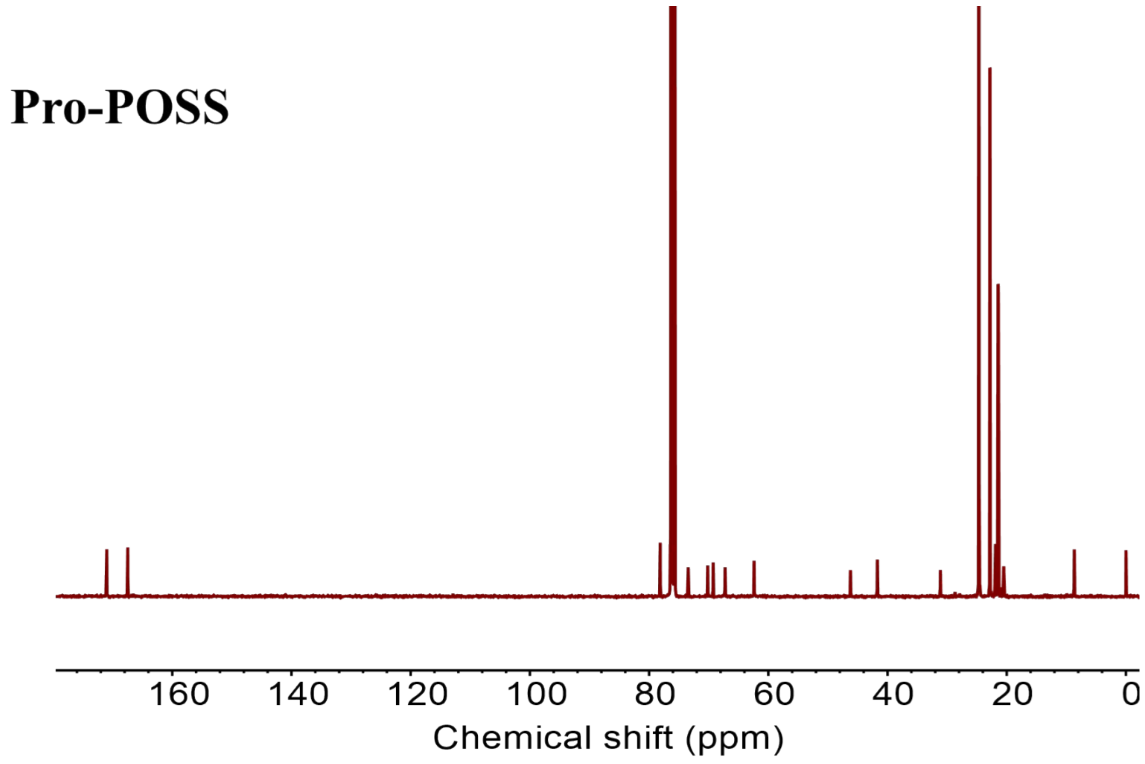


**Figure S15**. $^{13}C$ NMR spectrum of Pro-POSS.

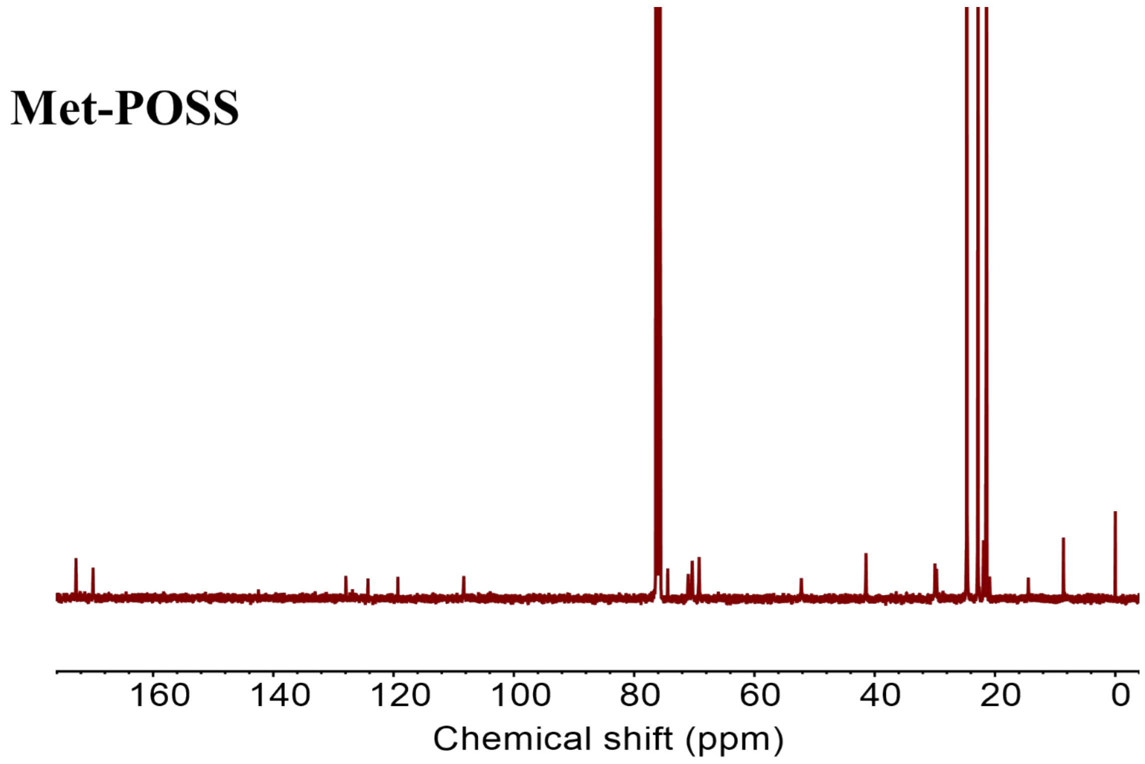


**Figure S16**. $^{13}C$ NMR spectrum of Met-POSS.

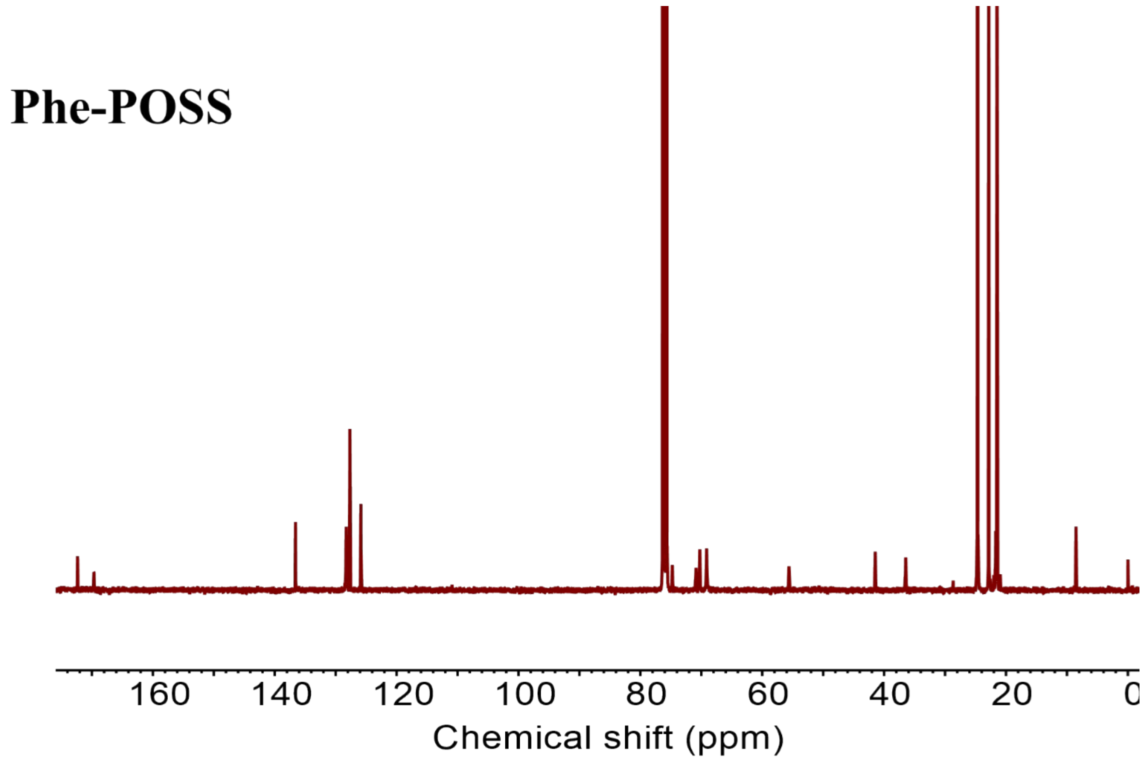


**Figure S17**. $^{13}C$ NMR spectrum of Phe-POSS.

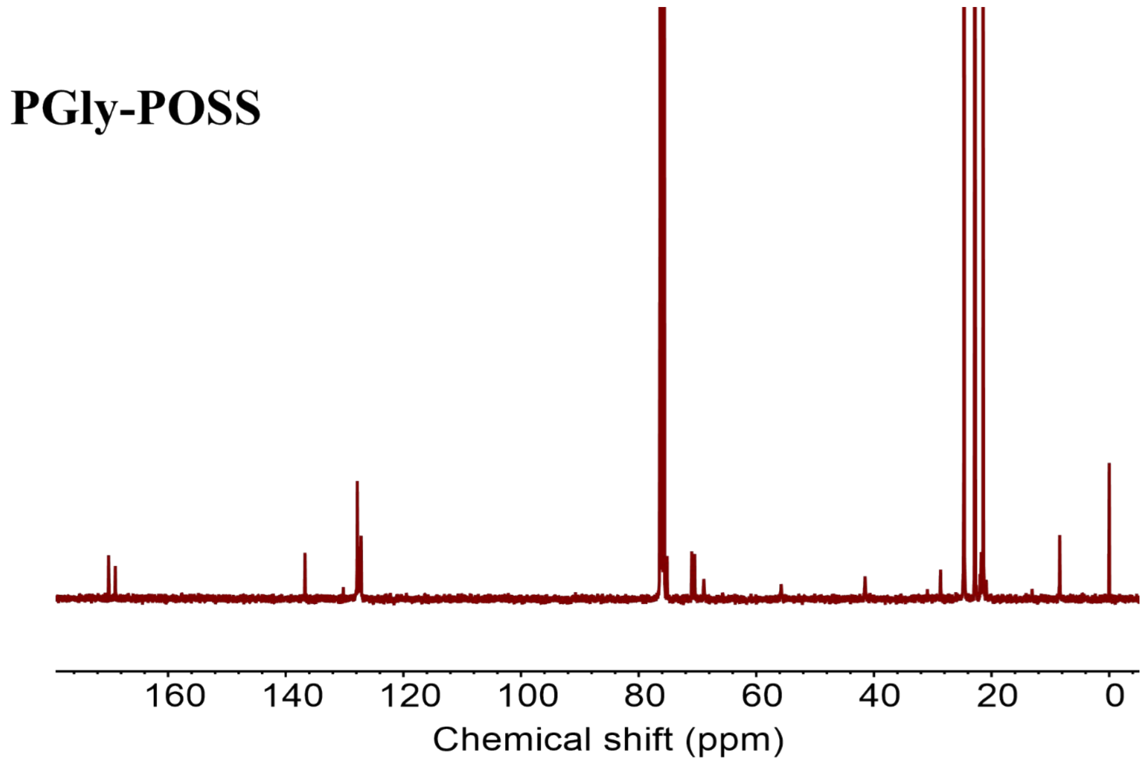


**Figure S18**. $^{13}C$ NMR spectrum of PGly-POSS.

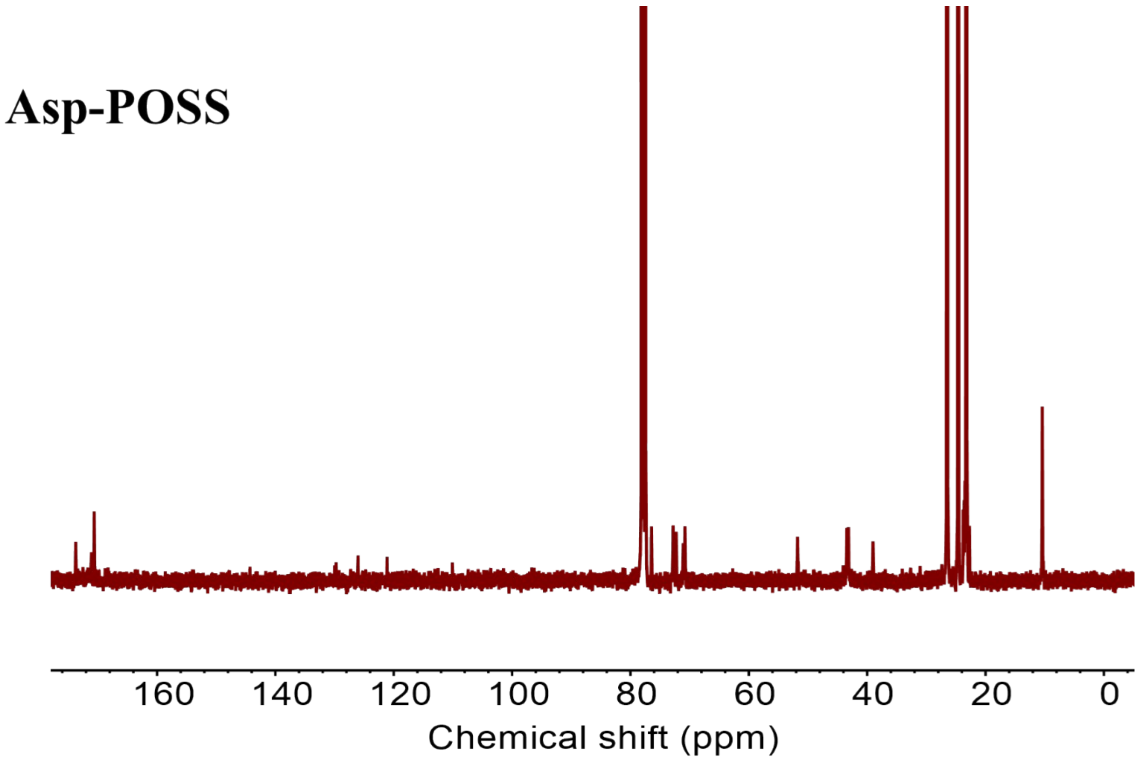


**Figure S19**. $^{13}C$ NMR spectrum of Asp-POSS.

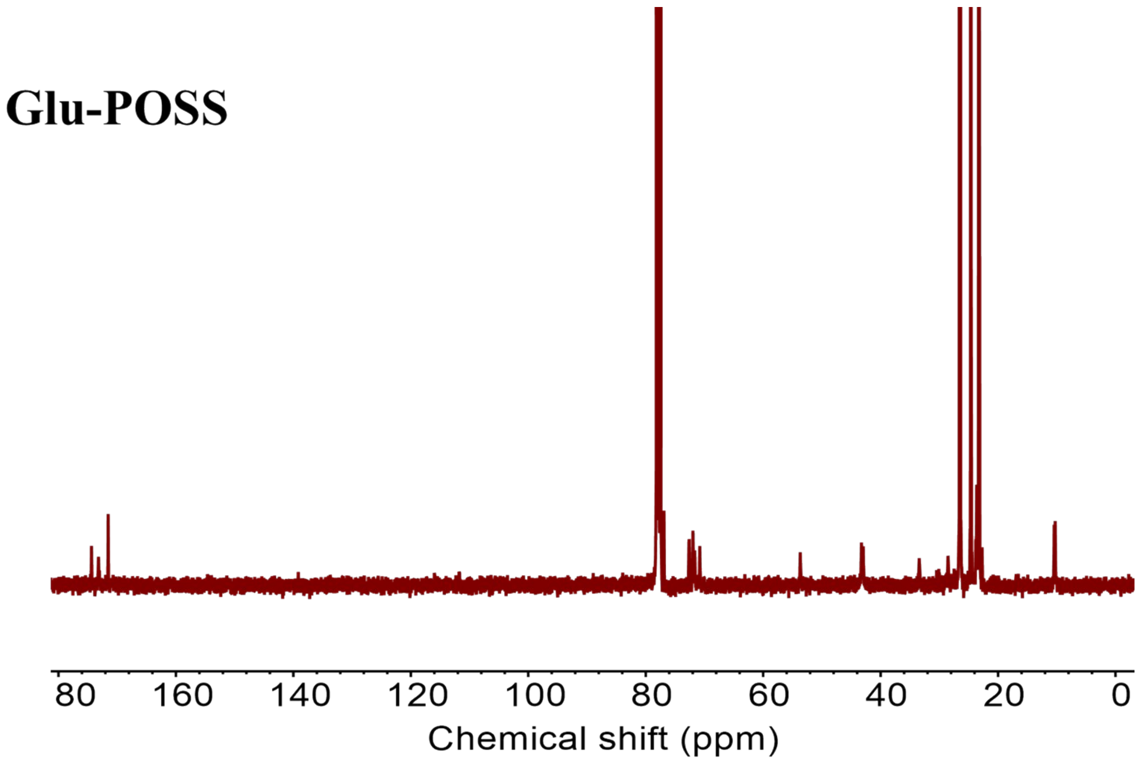


**Figure S20**. $^{13}C$ NMR spectrum of Glu-POSS.

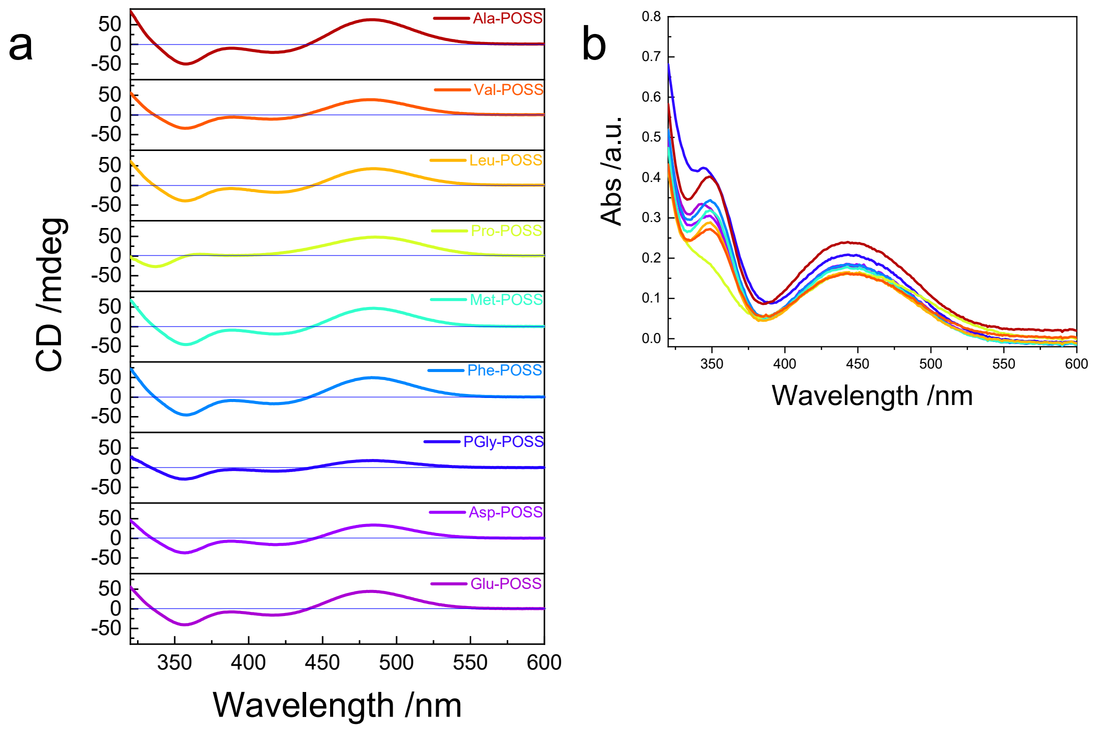


**Figure S21**. (a) CD spectra and (b) corresponding UV-vis spectra of POSS derivatives (1 mM, in $CHCl_3$)

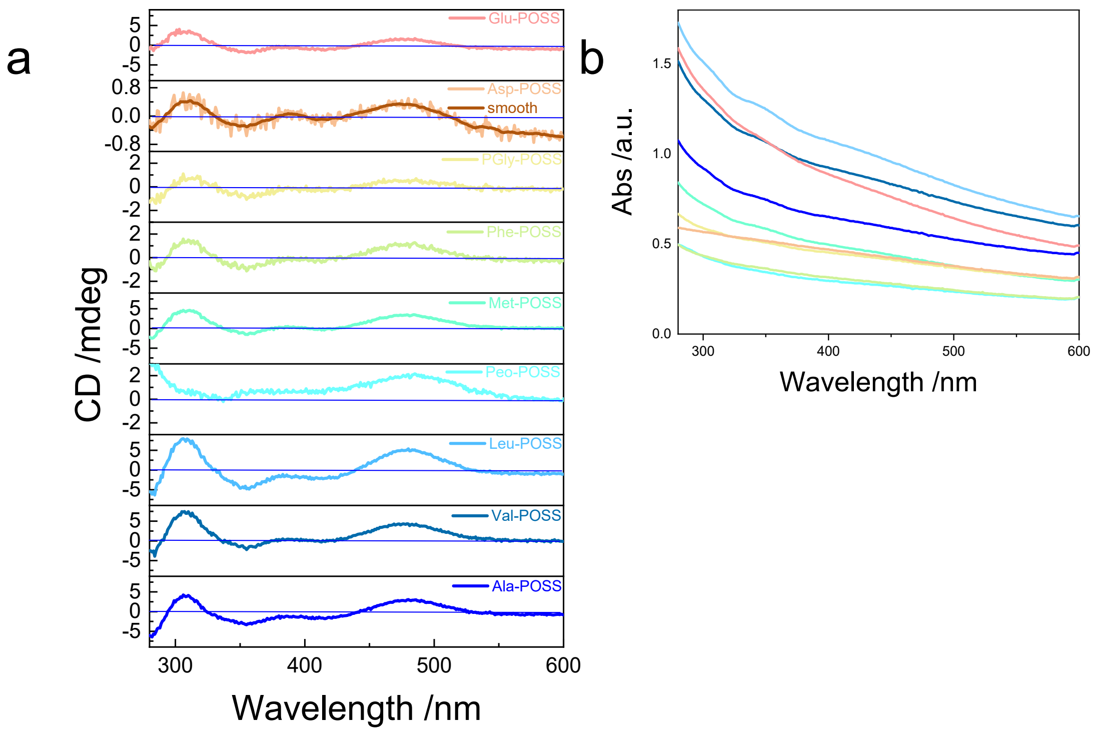


**Figure S22**. (a) Solid ECD spectra and (b) corresponding UV-vis spectra of POSS derivatives.

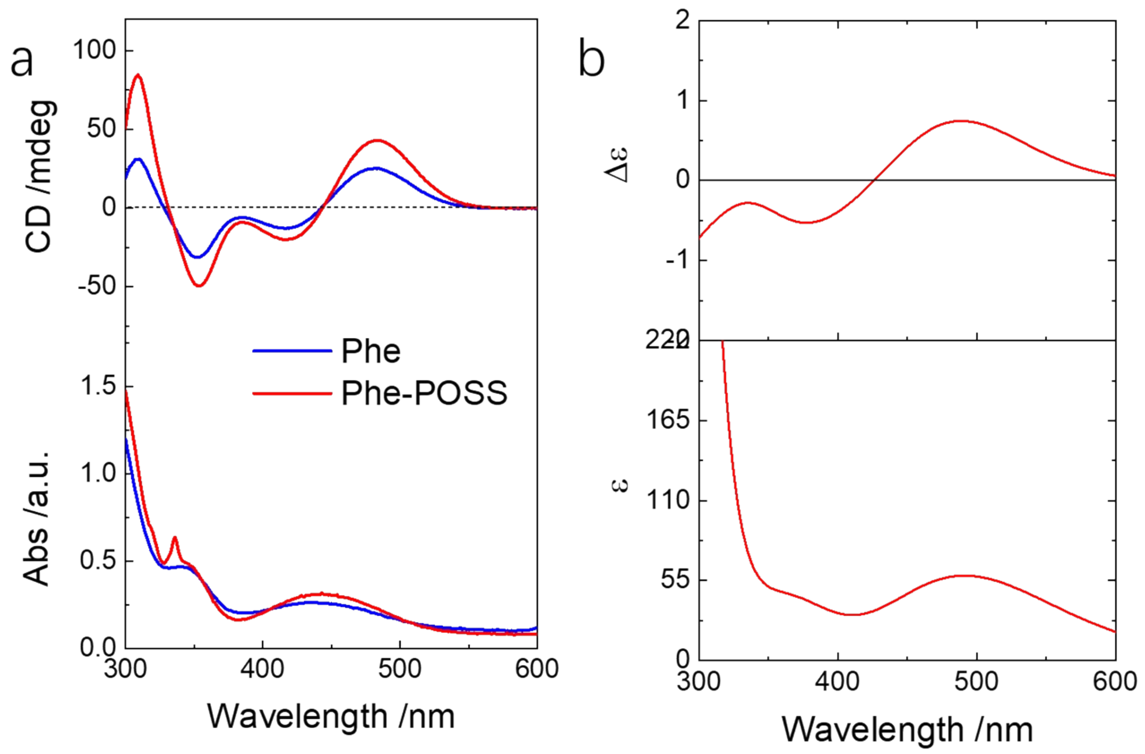


**Figure S23**. (a) Comparison of CD spectra between Phe (1,1'-ferrocenyl diphenylalanine) and Phe-POSS. (b) Calculated ECD spectra of Phe.

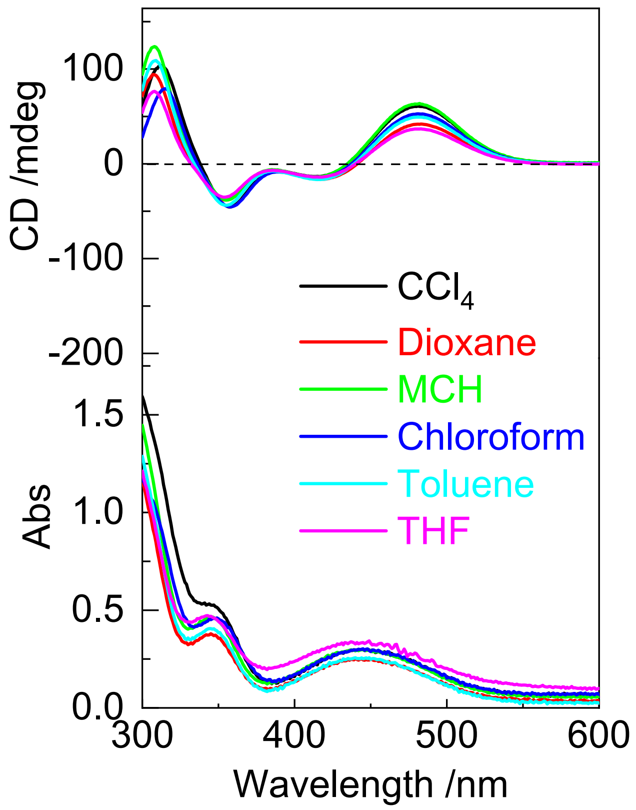


**Figure S24**. CD spectra of *L*-Val-POSS in different solvents (1 mM).

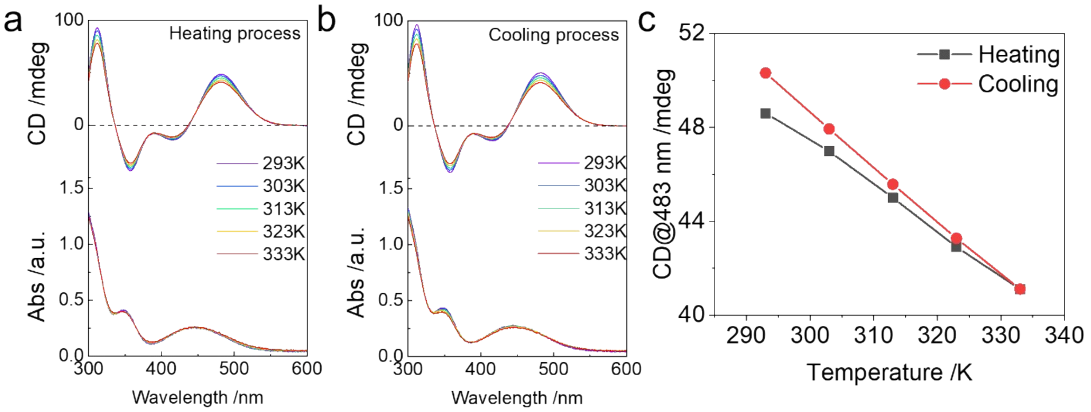


**Figure S25**. (a,b) Temperature-variable CD spectra of *L*-Val-POSS (1 mM) in the heating and cooling processes. (c) Corresponding CD intensity at 483 nm as a function of temperature.

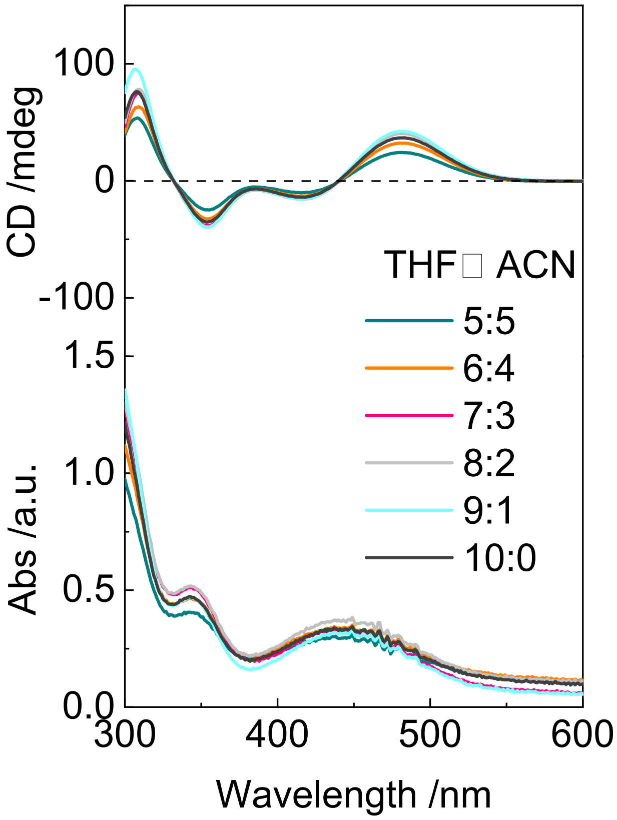


**Figure S26**. CD spectra of *L*-Val-POSS with different solvent ratios. When the fraction of ACN was higher than 50 vol%, precipitates shall be given. With increasing ACN fraction, no apparent correlation of CD signal and solvent fraction but irregularly changed Cotton effects were observed.

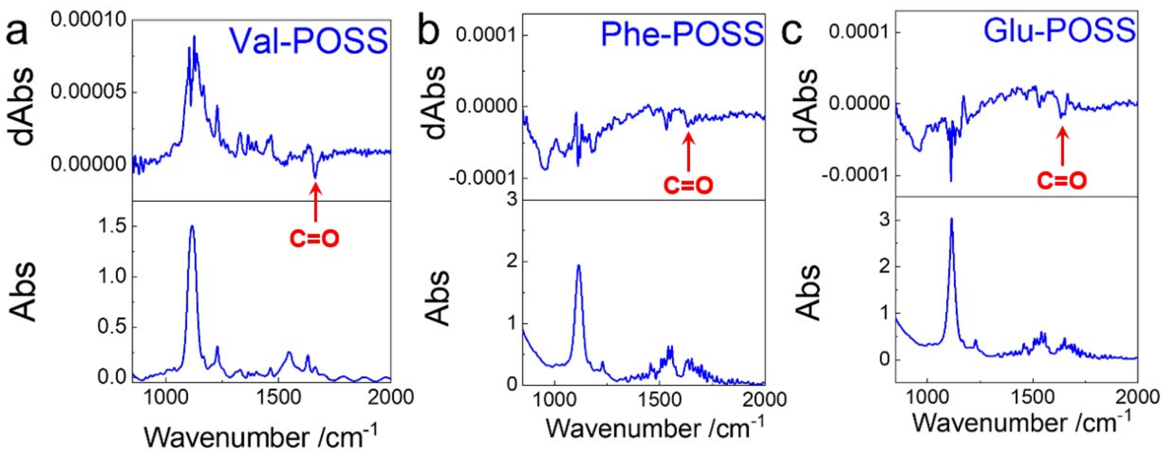


**Figure S27**. VCD spectra of (a) Val-POSS, (b) Phe-POSS, and (c) Glu-POSS (50 mg/mL in $CCl_4$).

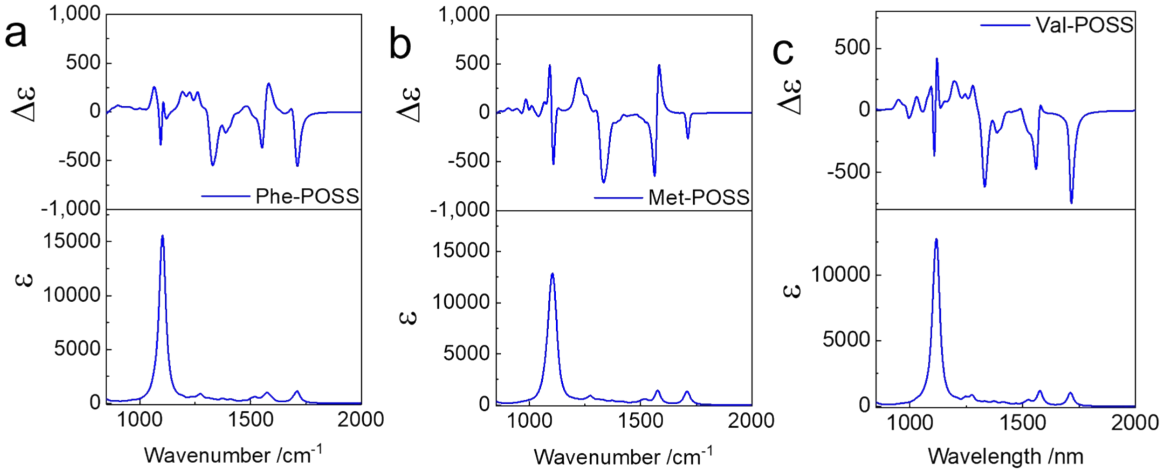


**Figure S28**. Simulated VCD spectra of (a) Phe-POSS, (b) Met-POSS and (c) Val-POSS (B3LYP/6-311g FWHM 15 $cm^{-1}$).

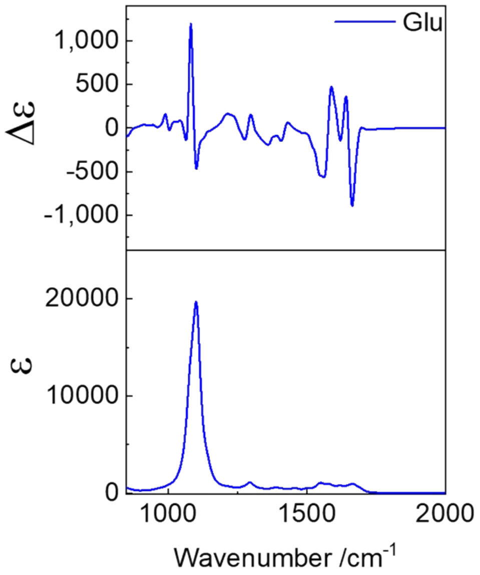


**Figure S29**. Simulated VCD spectra of Glu-POSS (B3LYP/6-31g FWHM 15 $cm^{-1}$).

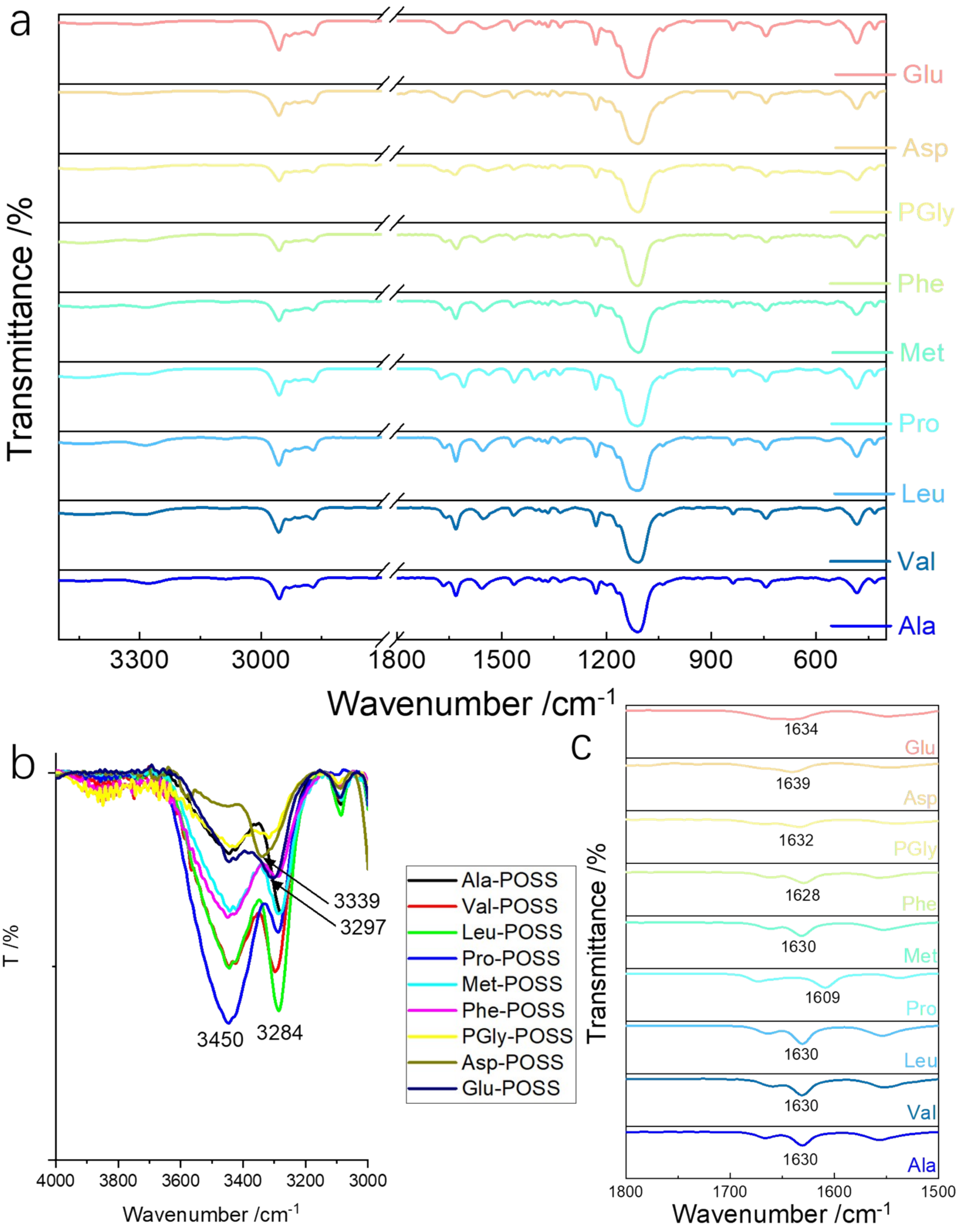


**Figure S30**. (a) FT-IR spectra of POSS derivatives. (b,c) Partial FT-IR spectra of each building units.

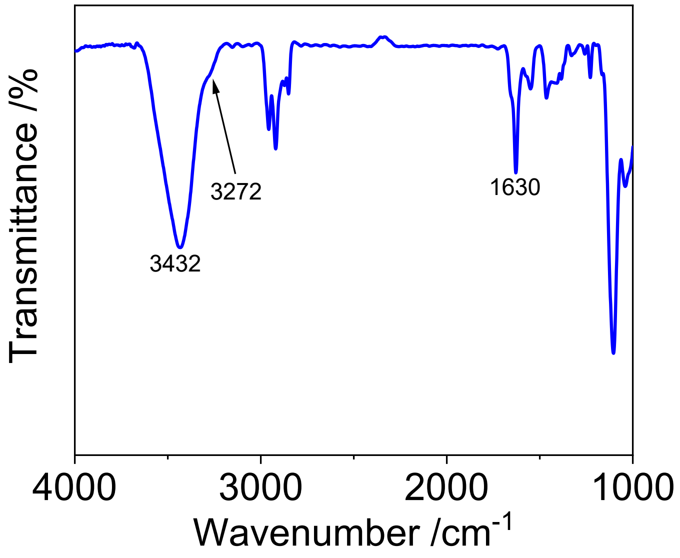


**Figure S31**. FT-IR spectrum of Val-POSS measured in liquid state (1 mM in $CCl_4$).

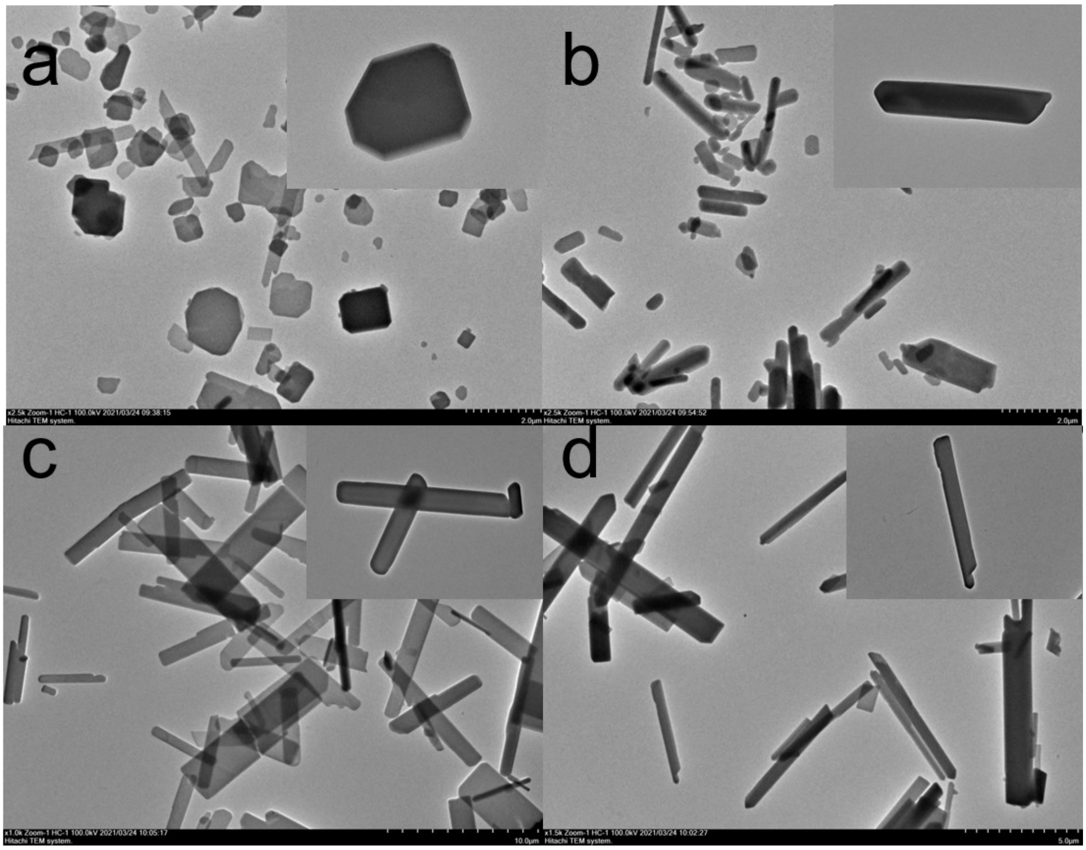


**Figure S32.** TEM image of (a) Ala-POSS, (b) Leu-POSS, (c) Phe-POSS, and (d) Met-POSS (1 mM, THF/MeCN 1/9).

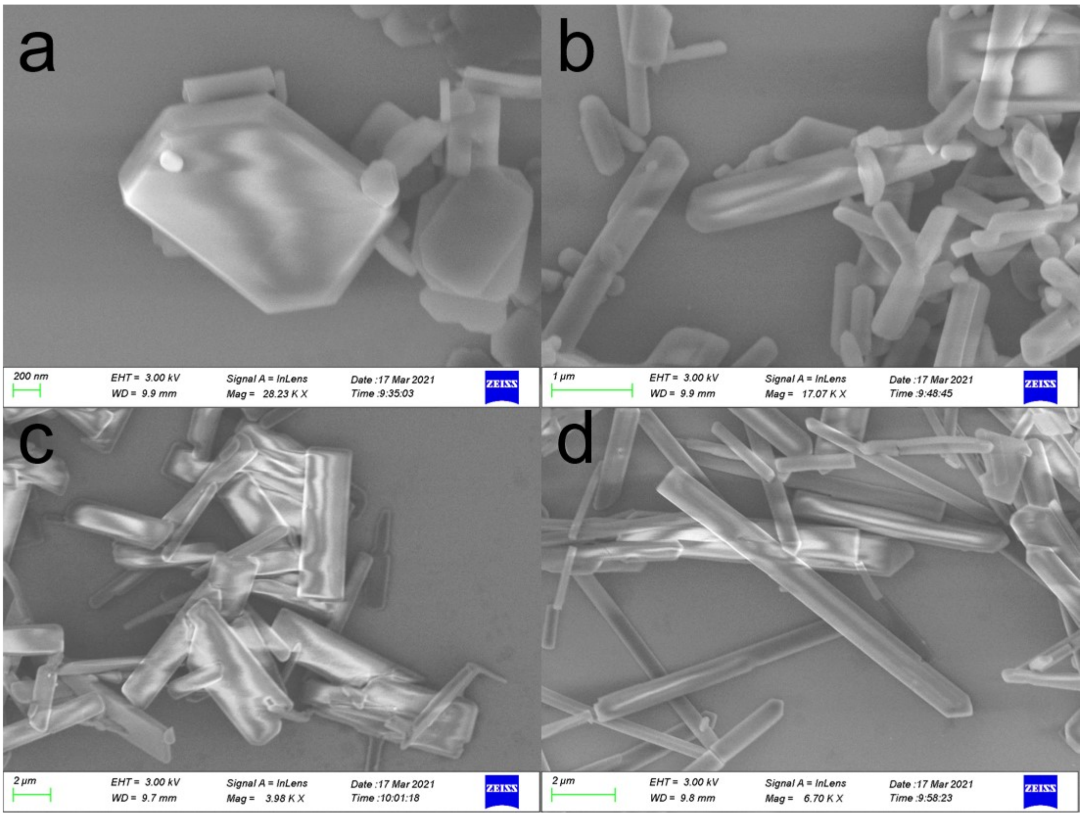


**Figure S33**. SEM image of (a) Ala-POSS, (b) Leu-POSS, (c) Phe-POSS, and (d) Met-POSS (1 mM, THF/MeCN 1/9).

**Table S1.** Crystal data of Phe-POSS.

| | |
|---|---|
| **Deposition Number** | 2096836 |
| **Formula** | $C_{92}$ $H_{166}$ Fe $N_4$ $O_{28}$ $Si_{16}$ |
| **Temperature(K)** | 293(2) |
| **Wavelength** | 1.54184Å |
| **Crystal system** | orthorhombic |
| **Space group** | P $2_1$ $2_1$ $2_1$ |
| **a,b,c/Å** | **a** 16.2474(5) **b** 17.2899(8) **c** 46.6858(17) |
| **V, Å3** | 13114.8 |
| **Cell angles** | **α** 90 **β** 90 **γ** 90 |
| **Z, Z'** | Z: 4 Z': 0 |
| **R-factor (%)** | 13.09 |

**Table S2.** Crystal data of Val-POSS.

| | |
|---|---|
| **Deposition Number** | 2096837 |
| **Formula** | $C_{84}$ $H_{166}$ Fe $N_4$ $O_{28}$ $Si_{16}$,2($C_4$ $H_8$ $O_2$) |
| **Temperature(K)** | 173.00(10) |
| **Wavelength** | 1.54184Å |
| **Crystal system** | monoclinic |
| **Space group** | P $2_1$ |
| **a,b,c/Å** | **a** 16.9205(5) **b** 17.5657(5) **c** 21.7093(5) |
| **V, Å3** | 6451.5 |
| **Cell angles** | **α** 90 **β** 90.980(2) **γ** 90 |
| **Z, Z'** | Z: 2 Z': 0 |
| **R-factor (%)** | 10.84 |

**Table S3.** Crystal data of Met-POSS.

| | |
|---|---|
| **Deposition Number** | 2096838 |
| **Formula** | $C_{80}$ $H_{156}$ Fe $N_4$ $O_{27}$ $S_2$ $Si_{16}$, $C_{71}$ $H_{136}$ Fe $N_4$ $O_{28}$ S $Si_{16}$, $C_2$ $H_6$ S |
| **Temperature(K)** | 173.01(10) |
| **Wavelength** | 1.54184Å |
| **Crystal system** | monoclinic |
| **Space group** | C2 |
| **a,b,c/Å** | **a** 49.9932(14) **b** 17.2234(3) **c** 37.4731(9) |
| **V, Å3** | 26797.5 |
| **Cell angles** | **α** 90 **β** 123.849(3) **γ** 90 |
| **Z, Z'** | Z: 8 Z': 0 |
| **R-factor (%)** | 13.41 |
